# Chromatin Central: towards the comparative proteome by accurate mapping of the yeast proteomic environment

**DOI:** 10.1186/gb-2008-9-11-r167

**Published:** 2008-11-28

**Authors:** Anna Shevchenko, Assen Roguev, Daniel Schaft, Luke Buchanan, Bianca Habermann, Cagri Sakalar, Henrik Thomas, Nevan J Krogan, Andrej Shevchenko, A Francis Stewart

**Affiliations:** 1 MPI of Molecular Cell Biology and Genetics, Pfotenhauerstrasse 108, 01307 Dresden, Germany; 2 Genomics, BioInnovationsZentrum, Technische Universität Dresden, Am Tatzberg 47-51, 01307 Dresden, Germany; 3 Department of Cellular and Molecular Pharmacology, University of California, San Francisco, 1700 4th Street, San Francisco, CA 94158, USA

## Abstract

**Background:**

Understanding the design logic of living systems requires the understanding and comparison of proteomes. Proteomes define the commonalities between organisms more precisely than genomic sequences. Because uncertainties remain regarding the accuracy of proteomic data, several issues need to be resolved before comparative proteomics can be fruitful.

**Results:**

The *Saccharomyces cerevisiae *proteome presents the highest quality proteomic data available. To evaluate the accuracy of these data, we intensively mapped a proteomic environment, termed 'Chromatin Central', which encompasses eight protein complexes, including the major histone acetyltransferases and deacetylases, interconnected by twelve proteomic hyperlinks. Using sequential tagging and a new method to eliminate background, we confirmed existing data but also uncovered new subunits and three new complexes, including ASTRA, which we suggest is a widely conserved aspect of telomeric maintenance, and two new variations of Rpd3 histone deacetylase complexes. We also examined the same environment in fission yeast and found a very similar architecture based on a scaffold of orthologues comprising about two-thirds of all proteins involved, whereas the remaining one-third is less constrained. Notably, most of the divergent hyperlinks were found to be due to gene duplications, hence providing a mechanism for the fixation of gene duplications in evolution.

**Conclusions:**

We define several prerequisites for comparative proteomics and apply them to examine a proteomic environment in unprecedented detail. We suggest that high resolution mapping of proteomic environments will deliver the highest quality data for comparative proteomics.

## Abstract 2

High resolution mapping of the proteomic environment and proteomic hyperlinks in fission and budding yeast reveals that divergent hyperlinks are due to gene duplications.

## Background

Understanding the design logic of living systems is now mainly based on genomics and DNA sequence comparisons. Typically, protein comparisons are evaluated by sequence alignments. However, living systems run programs that are written both as passive information (the genome) and as dynamic, molecular ecologies (the proteome). This dichotomy drives proteomic research because no living system can be solely described by its DNA sequence. Accurate proteomic maps are logically the next dataset required to complement complete genome sequences. However, the generation of reliable proteomic data remains challenging [1-4].

The budding yeast, *Saccharomyces cerevisiae*, has led eukaryotic research in several fields, particularly genomics, reverse genetics, cell biology and proteomics. For proteomic mapping, *S. cerevisiae *has been the main venue for the evaluation of various methodologies, which led to the clear conclusion that biochemical methods based on physiological expression levels deliver the most accurate results. In contrast, bioinformatic, yeast two hybrid and overexpression approaches generate less accurate data that require validation by a different means [1-4].

In contrast to a genome sequence, it is unlikely that a proteomic map can ever be complete because proteomes change in response to alterations of cellular condition. Proteomes include a very large number of post-translational modifications that are inherently variable, as well as protein-protein interactions that vary over a wide range of stabilities. Nevertheless, a proteome is based on a stable core of protein complexes, which can be accurately mapped by biochemical approaches [2]. Hence, an accurate proteomic map will be based on the constellation of stable protein complexes for a given cellular condition. The map then provides a scaffold onto which transient interactions and post-translational modifications can be organized. Thereby, proteomes can be rationalized [5,6].

The quest to understand proteomes has led to the definition of new perspectives and terms, such as a proteomic 'environment', which describes the local relationships within a group of interacting proteins; 'hubs', which is applied to proteins that interact with many other proteins [2]; and 'hyperlinks', which is a term we applied to proteins that are present in more than one stable protein complex [7]. Similarly, insight into proteomes can be gleaned from comparative proteomics [8]. However, without accurate proteomic maps, these new terms and perspectives, particularly those derived from comparative proteomics, have limited meaning.

To map the budding yeast proteome accurately, methodologies for physiological expression and purification of tagged proteins were developed based on gene targeting with the tandem affinity purification (TAP) tag [9,10]. The high throughput application of these methods by two different groups led to the best proteomic map datasets for any cell, whether prokaryotic or eukaryotic [11,12]. Collins *et al. *consolidated both datasets into one of even higher quality; nevertheless, they recommended more intensely focused data gathering to evaluate accuracy [13].

Here we address the issue of proteomic accuracy by intense exploration of a section of the budding yeast proteome that is related to chromatin regulation. Chromatin is regulated by multiprotein complexes, which dynamically target nucleosomes with a multitude of reversible modifications, such as acetylation, methylation, phosphorylation and ubiquitination (reviewed in [14]). Also, in budding yeast, many of these complexes have been individually isolated and functionally characterized, which provides a rich and detailed source of reference information. Previously, we concluded that greater accuracy can be attained by sequential tagging to reciprocally validate interactions [10,15,16]. Sequential tagging of candidate interactors to map a proteomic environment has also been termed proteomic navigation or SEAM (short for Sequential rounds of Epitope tagging, Affinity isolation and Mass spectrometry). For a low throughput approach, which also permits a more intense focus on individual experiments, sequential tagging will deliver improvements in accuracy.

Several other factors may reduce mapping accuracy. In the *S. cerevisiae *proteome every fourth protein is apparently a proteomic hyperlink [5]. That is, a member of more than one distinct protein complex. Hence, many pull-downs are mixtures of completely or partially co-purified complexes, together with other sub-stoichiometric and pair-wise interactors. Also, sorting out background proteins from genuine interactors remains challenging [5,17-19], especially when proteins are identified by mass spectrometric techniques with enhanced dynamic range, such as liquid chromatography tandem mass spectrometry (LC-MS/MS) or LC matrix-assisted laser desorption/ionization mass spectrometry (MALDI) MS/MS, which produce a large number of confident protein identifications in each pull-down. Furthermore, until recently, mass spectrometric identifications have mostly neglected the quantitative aspect. It was (and, largely, still is) difficult to determine which proteins are *bona fide *members of a tagged complex and, therefore, stoichiometric, and which interactors are sub-stoichiometric. Here we address these issues to develop refinements for improved accuracy of mapping, including working criteria to identify common background proteins and stoichiometric interactors.

Using the sequential strategy and these refinements, we mapped a large proteomic environment that we term 'Chromatin Central' because it includes eight protein complexes interconnected by hyperlinks encompassing the major histone aceytyltransferases and deacetylases in budding yeast. As evidence for mapping accuracy, we made several discoveries, including the identification of new subunits of known complexes and new complexes.

To exploit the quality of the map for comparative proteomics, we then explored the same proteomic environment in the distantly related yeast *Schizosaccharomyces pombe*. This enabled a detailed comparison of two highly accurate proteomic environments to shed light on the evolution of proteomic architecture.

## Results

### Establishing a proteomic environment

**Figure 1:**
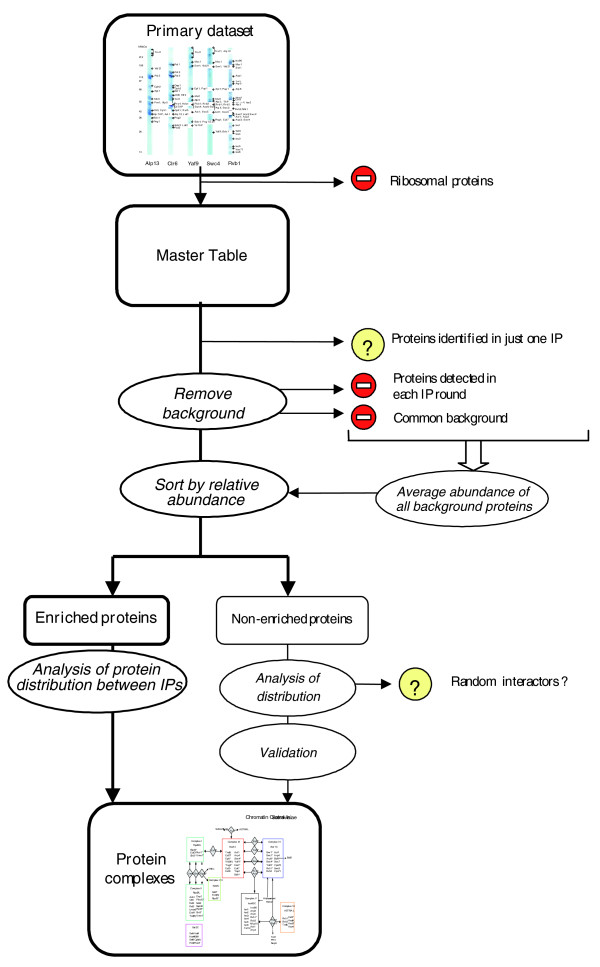
Data processing workflow. The primary dataset is a complete list of proteins identified in IP experiments that were used to map the Chromatin Central proteomic environment in any of the two yeasts. After removal of ribosomal proteins, all hits together with their A-indices were compiled into a non-redundant master table and grouped according to IP rounds. To accurately determine the scaffold protein complexes, we further removed from the master table proteins having A-index = 1 that were identified only in one IP experiment and common background proteins. Using the average A-index of background proteins as a selection threshold, the remaining proteins were sorted into two large groups: proteins enriched in corresponding IP experiments and proteins whose abundance remained at the background level. Proteins in the first group were considered as genuine interactors and were assigned to complexes, assuming IP experiments in which they were identified. From the second group, only proteins that were validated by a reciprocal IP experiment were assigned to the corresponding complexes.

Our approach to charting proteomic environments relies upon the sequential use of TAP and mass spectrometry to identify stable protein assemblies. In a typical TAP pull-down experiment, LC-MS/MS analysis identified over 500 proteins containing stoichiometric and transient *bona fide *protein interactors, along with a large number of background proteins of diverse origin and abundance. To dissect the composition of complexes, we employed a layered data mining approach. First, we sorted out common background proteins and then distinguished proteins specifically enriched in the TAP isolation using semi-quantitative estimates of their abundance (Figure 1).

#### Common background proteins

A list was established based on background proteins from proteins repetitively found in 20 diverse immunoaffinity purifications (IPs) that were selected from three unrelated projects, this project being one of those three. The other two were based on mitotic cell cycle regulation and vesicle transport. The tagged proteins and their known interactors, as well as ribosomal proteins, were first removed from the 20 primary IP lists. Then, of more than 2,000 proteins identified in these 20 IPs, 119 (Table S1 in Additional data file 1) were defined as common background because they were found at least once in each of the three independent projects. This list of 119 includes proteins with molecular weights ranging from 11 to 250 kDa and expression levels of 100 to 10^6 ^molecules per cell [20,21]. Most of these common background proteins were cytoplasmic [21-23], including heat shock, translation factors and abundant housekeeping enzymes. Once these common background proteins were removed from a particular IP list, it was further refined using abundance index (A-index) filtering.

#### Index of relative abundance

The absolute amounts of immunoprecipitated protein varies between TAP purifications. However, within a purification, members of a stable protein complex should be isolated in approximately stoichiometric amounts and relatively enriched compared to the other detected proteins. Abundant background proteins are an exception; however, we always removed them from the list at the very beginning of the data processing routine as described above.

To estimate the relative abundance of individual proteins and hence obtain an additional means to distinguish genuine interactors from background, we used an arbitrary A-index. It was calculated as a ratio of the total number of MS/MS spectra acquired for a given protein (reported as 'matched queries' for each MASCOT hit) to the number of unique peptide sequences they matched. Essentially, the A-index is a relative measure of the amounts of co-isolated proteins from the gel. We applied it as a convenient way to distinguish *bona fide *subunits of the tagged complex from background proteins because they should be relatively enriched, compared to background. In a series of preliminary experiments, we observed that the A-index monotonously increased with increasing amount of loaded proteins from 50 to 800 fmols. When determined for six standard proteins of various molecular weights and properties, the A-index varied within a 50% margin at any given protein loading (Figure S1 in Additional data file 2).

#### Selecting genuine interactions to determine protein complexes

Each protein complex was isolated several times within a round of IP experiments that used different baits [10,15,16]. Hence, several independent IPs established the protein complex composition or identified a hyperlink to another protein assembly (Figure S2 in Additional data file 2). In turn, proteins co-purified with a hyperlink and that did not belong to the complex characterized in the current round were selected as baits for the next sequential round. For *S. cerevisiae*, within five IP rounds, 21 out of 26 pull downs from unique baits were successful (for the full list of identified proteins, see Table S2 in Additional data file 1). After the ribosomal proteins were removed, a non-redundant list of proteins identified in all IPs, together with their A-indices, was assembled into a master table containing 1,301 proteins in total (Table S3 in Additional data file 1). Then we removed common background proteins and low abundant proteins whose A-indices were equal to 1 and were identified only once in the total of 21 IPs.

The common background proteins listed in the master table had an average A-index value of 1.4. We noticed that A-indices of more than 90% of background proteins were within 25% of the average, so we employed this empirical threshold to further sort out experiment-specific background. Since genuine interactors were supposed to be enriched in the IPs compared to background proteins, we introduced an arbitrary cut-off of 1.75 for A-indices of genuine protein interactions (Table S3 in Additional data file 1).

Proteins were recognized as stoichiometric core members of complexes if they did not belong to common background, were specifically enriched in corresponding IPs, and, most importantly, were co-isolated with baits within the corresponding round of sequential IPs (Figure 1). Potentially, these criteria might have eliminated some transient (yet genuine) interactors; however, we placed our priorities upon accuracy. Although the chosen 25% margin might look arbitrary, the entire approach was validated by a good concordance of the composition of protein complexes in *S. cerevisiae *Chromatin Central with the published evidence, as described below.

### Chromatin Central in *S. cerevisiae*

**Figure 2:**
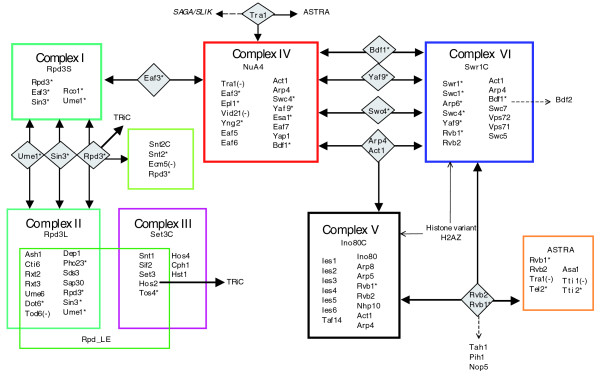
Chromatin Central proteomic environment in *S. cerevisiae*. Individual protein complexes are boxed; TAP-tagged subunits are indicated with asterisks. The proteomic hyperlinks (proteins shared between the individual complexes) are shown between the complexes in grey diamonds. The hyperlink from Tra1 to the SAGA/SLIK complex is designated with a dashed line/filled arrow because it was not identified in this work, but inferred from published evidence. Gene names designated with a minus (-) symbol indicate that their TAP tagging/immunoaffinity purification failed. Several relatively abundant (A-index > 1.75) pair-wise interactors, also identified in proteome-wide screens [101,102], are mapped onto the scheme (dashed line/unfilled arrow). Set3C complex was previously characterized by TAP-tagging method in [10].

From 1,301 unique open reading frames (ORFs) in the master table, only 63 proteins (less than 5% of all identified proteins) matched the above selection criteria, comprising 9 stable protein complexes connected by 12 proteomic hyperlinks. Three out of these nine (ASTRA (for ASsembly of Tel, Rvb and Atm-like kinase), Snt2C and Sc_Rpd-LE (for Rpd3L expanded with Set3C core); Figure 2) are reported here for the first time, whereas the other six (complexes I-VI) have been characterized previously (note that the prefixes Sc_ and Sp_ refer to proteins from *S. cerevisiae *and *S. pombe*, respectively; the suffix 'C' always refers to the protein complex).

Chromatin Central comprised four distinct protein assemblies, including: the histone deacetylase Rpd3p (Sc_Rpd3S, Sc_Rpd3L [24,25], Sc_Rpd-LE and Sc_Snt2C); at least two histone acetyltransferase complexes, Sc_NuA4 [26] and SAGA/SLIK [27]; and two ATP-dependent chromatin remodeling complexes, Sc_Swr1C and Sc_Ino80C [28,29]. The compositions of the individual protein complexes (Tables 1, 2, 3, 4, 5) were compared with previous reports. Surprisingly, we found some discrepancies with data from the best proteome maps even though they were also obtained by TAP tagging [11,12]. In contrast, our results agree with several publications describing the biochemical and functional characterization of the individual complexes. In particular, complexes I, V and VI are identical to the previously reported Sc_Rpd3S, Sc_Swr1C and Sc_INO80C, respectively [24,25,28,29].

**Table 1:** Members of NuA4 histone acetylase complexes in the Chromatin Central proteomic environment

*S. cerevisiae*			*S. pombe*			Sequence comparison	
		
Gene name	ORF	MW (kDa)	Gene name	ORF	MW (kDa)	Identity/similarity (%)	Orthologue
*TRA1*	YHR099W	433	*TRA2*	SPAC1F5.11c	420	33/53	Gene duplication
*VID21*	YDR359C	112	*VID21*	SPCC1795.08c	112	23/40	
*EPL1*	YFL024C	97	*EPL1*	SPCC830.05c	65	36/51	
*ARP4*	YJL081C	55	*ALP5*	SPBP23A10.08	49	35/51	
*SWC4*	YGR002C	55	*SWC4*	SPAC9G1.13c	47	30/44	
*ESA1*	YOR244W	52	*MST1*	SPAC637.12c	54	56/71	
*YAF9*	YNL107W	26	*YAF9*	SPAC17G8.07	25	45/64	
*ACT1*	YFL039C	42	*ACT1*	SPBC32H8.12c	42	90/97	
*EAF3*	YPR023C	45	*ALP13*	SPAC23H4.12	39	32/47	
*YNG2*	YHR090C	32	*PNG1*	SPAC3G9.08	31	32/53	
*EAF7*	YNL136W	49	*EAF7*	SPBC16A3.19	31	22/43	
*YAP1*	YML007W	72	*PAP1*	SPAC1783.07c	62	26/41	
*EAF5*	YEL018W	32					No orthologues in *S. pombe*
*EAF6*	YJR082C	13					Predicted orthologue SPAC6F6.09
*BDF1*	YLR399C	77					Predicted orthologue SPCC1450.02
			*BDC1*	SPBC21D10.10	34		No orthologues in *S. cerevisiae*

**Table 2:** Members of histone deacetylase complexes of the Chromatin Central proteomic environments

	*S. cerevisiae*			*S. pombe*			Sequence comparison	
			
	Gene name	ORF	MW (kDa)	Gene name	ORF	MW (kDa)	Identity/similarity (%)	Orthologue
Rpd3S/Clr6S	*RPD3*	YNL330C	49	*CLR6*	SPBC36.05C	46	67/82	
complexes	*SIN3*	YOL004W	175	*PST2*	SPAC23C11.15	125	24/41	Gene duplication
	*RCO1*	YMR075W	79	*CPH2*	SPAC2F7.07c	69	26/44	
	*RCO1*	*YMR075W*	79	*CPH1*	SPAC16C9.05	45	25/42	Gene duplication
	*EAF3*	YPR023C	45	*ALP13*	SPAC23H4.12	39	32/47	
	*UME1*	YPL139C	51					Functional orthologue of prw1
				*PRW1*	SPAC29A4.18	48		Functional orthologue of ume1

Rpd3L/Clr6L	*RPD3*	YNL330C	49	*CLR6*	SPBC36.05C	46	67/82	
complexes	*SIN3*	YOL004W	175	*PST1*	SPBC12C2.10C	171	32/49	
	*SIN3*	*YOL004W*	175	*PST3*	SPBC1734.16C	133	27/44	Gene duplication
	*CTI6*	YPL181W	57	*CTI6*	SPBC1685.08	46	28/44	
	*PHO23*	YNL097C	37	*PNG2*	SPBC1709.11c	35	29/45	
	*RXT3*	YDL076C	34	*RXT3*	SPCC1259.07	39	28/40	
	*RXT2*	YBR095C	49	*RXT2*	SPBC428.06c	27		Figure S3
	*SDS3*	YIL084C	38	*SDS3*	SPAC25B8.02	31		
	*DEP1*	YAL013W	48	*DEP1*	SPBC21C3.02c	55		Figure S3
	*SAP30*	YMR263W	23					No orthologues in *S. pombe*
	*UME6*	YDR207C	91					No orthologues in *S. pombe*
	*DOT6*	YER088C	72					No orthologues in *S. pombe*
	*TOD6*	YBL054W	59					No orthologues in *S. pombe*
	*ASH1*	YKL185W	66					No orthologues in *S. pombe*
	*UME1*	YPL139C	51					Functional orthologue of prw1
				*PRW1*	SPAC29A4.18	48		Functional orthologue of ume1
				*LAF1*	SPAC14C4.12c	34		Predicted orthologues YAL034C* and YOR338W
				*LAF2*	SPCC1682.13	31		Predicted orthologues YAL034C* and YOR338W

Snt2 complex	*SNT2*	YGL131C	163					Predicted orthologue SPAC3H1.12c
	*ECM5*	YMR176W	163					No orthologues in *S. pombe*
	*RPD3*	YNL330C	49					SPBC36.05c

Set3 Complex	*SNT1*	YCR033W	138	*SNT1*	SPAC22E12.19	75	25/44	
	*HOS2*	YGL194C	51	*HDA1*	SPAC3G9.07c	49	59/76	
	*SIF2*	YBR103W	59	*HIF2*	SPCC1235.09	63	22/41	
	*SET3*	YKR029C	85	*SET3*	SPAC22E12.11c	95	24/42	
	*HOS4*	YIL112W	124					No orthologues in *S. pombe*
	*CPH1*	YDR155C	17					Predicted orthologue SPBC28F2.03*
	*HST1*	YOL068C	58					Predicted orthologue SPBC16D10.07c
	*TOS4*	YLR183C	55					Predicted orthologue SPAP14E8.02

*Detected with related bait(s) as a minor component (Table S4 in Additional data file 1).

**Table 3:** Members of chromatin remodeling complexes of the Chromatin Central proteomic environment

	*S. cerevisiae*			*S. pombe*			Sequence comparison	
			
	Gene name	ORF	MW (kDa)	Gene name	ORF	MW (kDa)	Identity/similarity (%)	Orthologue
Swr1	*SWR1*	YDR334W	174	*SWR1*	SPAC11E3.01c	149	43/60	
complex	*SWC2*	YDR485C	90	*SWC2*	SPBP35G2.13C	36	24/45	
	*BDF1*	YLR399C	77	*BDF1*	SPCC1450.02	65	30/50	
	*SWC4*	YGR002C	55	*SWC4*	SPAC9G1.13c	47	30/44	
	*ARP4*	YJL081C	53	*ALP5*	SPBP23A10.08	49	35/51	
	*RVB1*	YDR190C	50	*RVB1*	SPAPB8E5.09	50	70/84	
	*RVB2*	YPL235W	51	*RVB2*	SPBC83.08	51	70/86	
	*ARP6*	YLR085C	50	*ARP6*	SPCC550.12	45	32/50	
	*SWC5*	YBR231C	34	*SWC5*	SPCC576.13	25	25/49	
	*VPS71*	YML041C	30	*VPS71*	SPBC29A3.05	16	30/45	
	*YAF9*	YNL107W	26	*YAF9*	SPAC17G8.07	25	45/64	
	*ACT1*	YFL039C	42	*ACT1*	SPBC32H8.12c	42	90/97	
	*HTZ1*	YOL012C	14	*PHT1*	SPBC11B10.10c	19	70/81	
	*SWC3*	YAL011w	73	*SWC3*	SPAC4H3.02c	45		Figure S3
	*SWC7*	YLR385c	15					No orthologues in *S. pombe*
				*MSC1*	SPAC343.11c	180		No orthologues in *S. cerevisiae*

INO80	*INO80*	YGL150C	171	*INO80*	SPAC29B12.01	183	45/60	
complex	*ARP8*	YOR141C	100	*ARP8*	SPAC664.02c	70	29/48	
	*ARP5*	YNL059C	88	*ARP5*	SPBC365.10	82	39/61	
	*RVB1*	YDR190C	50	*RVB1*	SPAPB8E5.09	50	70/84	
	*RVB2*	YPL235W	51	*RVB2*	SPBC83.08	51	70/86	
	*ARP4*	YJL081C	53	*ALP5*	SPBP23A10.08	49	35/51	
	*HTZ1*	YOL012C	14	*PHT1*	SPBC11B10.10c	19	70/81	
	*IES6*	YEL044W	19	*IES6*	SPAC222.04c	13	40/55	
	*IES2*	YNL215W	36	*IES2*	SPAC6B12.05c	34		Figure S3
	*IES4*	YOR189W	13	*IES4*	SPAC23G3.04	21		Figure S3
	*ACT1*	YFL039C	42	*ACT1*	SPBC32H8.12c	42	90/97	
	*TAF14*	YPL129w	27					Predicted orthologue SPAC22H12.02*
	*IES1*	YFL013C	79					No orthologues in *S. pombe*
	*IES3*	YLR052W	28					No orthologues in *S. pombe*
	*IES5*	YER092W	14					No orthologues in *S. pombe*
	*NHP10*	YDL002C	24					Predicted orthologues SPBC28F2.11 and SPAC57A10.09c
				*NHT1*	SPAC10F6.08c	38		No orthologues in *S. cerevisiae*
				*IEC1*	SPAC144.02	28		No orthologues in *S. cerevisiae*
				*IEC3*	SPCC1259.04	18		No orthologues in *S. cerevisiae*
				*IEC5*	New sequence, [GenBank:FJ493251]	17		No orthologues in *S. cerevisiae*

*Detected with related bait(s) as a minor component (Table S4 in Additional data file 1).

**Table 4:** Members of ASTRA complexes of the Chromatin Central proteomic environment

*S. cerevisiae*			*S. pombe*			Sequence comparison	
		
Gene name	ORF	MW (kDa)	Gene name	ORF	MW (kDa)	Identity/similarity (%)	Orthologue
*TRA1*	YHR099W	433	*TRA1*	SPBP16F5.03c	422	34/54	
*TTI1*	YKL033W	119	*TTI1*	SPCC622.13c	125	21/41	
*TEL2*	YGR099W	79	*TEL2*	SPAC458.03	99	23/43	
*RVB1*	YDR190C	50	*RVB1*	SPAPB8E5.09	50	70/84	
*RVB2*	YPL235W	51	*RVB2*	SPBC83.08	51	70/86	
*TTI2*	YJR136C	49	*TTI2*	SPBC1604.17c	53	23/46	
*ASA1*	YPR085c	51	*ASA1*	SPAC1006.02	41		Figure S3

**Table 5:** Other members of the Chromatin Central proteomic environment

	*S. cerevisiae*			*S. pombe*			Sequence comparison
			
	Gene name	ORF	MW (kDa)	Gene name	ORF	MW (kDa)	Identity/similarity (%)
TriC chaperonin-containing complexes	*CCT1*	YDR212w	60	*CCT1*	SPBC12D12.03	60	77/89
	*CCT2*	YIL142w	57	*CCT2*	SPAC1D4.04	57	69/83
	*CCT3*	YJL014W	59	*CCT3*	SPBC1A4.08c	58	69/83
	*CCT4*	YDL143w	58	*CCT4*	SPBC106.06	57	67/83
	*CCT5*	YJR064W	62	*CCT5*	SPAC1420.02c	59	64/82
	*CCT6*	YDR188w	60	*CCT6*	SPBC646.11	59	60/76
	*CCT7*	YJL111W	60	*CCT7*	SPBC25H2.12c	61	68/83
	*CCT8*	YJL008c	62	*CCT8*	SPBC337.05c	60	53/73

Selected stoichiometric pair-wise Interactors*	*BDF2*	YDL070W	72				
	*PIH1*	YHR034C	40				
	*TAH1*	YCR060W	12				
	*NOP5*	YOR310C	60	*NOP5*	SPAC23G3.06	57	
				*NAP11*	SPCC364.06	44	
				*NAP12*	SPBC2D10.11c	43	
				*KAP114*	SPAC22H10.03c	111	
				*CBF5*	SPAC29A4.04C	53	

*Corroborates previous publications [13,42,101,102].

In addition to the 12 known members of Sc_Rpd3L (complex II) [24,25], we identified 2 novel subunits, including the 72 kDa protein Sc_Dot6p (ORF name YER088C) and its 59 kDa homolog Sc_Tod6p (Twin of the Dot6; ORF name YBL054W). Their sequences share 31% identity; 46% similarity and both possess the chromatin specific SANT domain [30]. Furthermore, the involvement of Sc_Dot6 in the regulation of telomere silencing has been indicated [31].

In addition to the 14 known members of Sc_NuA4 (complex IV) [26,32], another new protein, the 72 kDa Sc_Yap1p (ORF name YML007W), which is a member of a family of fungal specific transcriptional activators, was identified as a subunit. Within Sc_Set3C (complex III) [10] we also identified a new member, the 55 kDa protein Sc_Tos4p (ORF name YLR183C). It is a putative transcription factor of the forkhead family. Tagging Sc_Tos4p pulled down the entire Sc_Set3C, except for the hyperlink Sc_Hst1p [5] (also, see Figure S2 in Additional data file 2 and Table S2 in Additional data file 1).

We identified 12 proteomic hyperlinks in Chromatin Central (Figure 2). One of these proteins, the 422 kDa Sc_Tra1p (ORF name YHR099W) is a core member of Sc_NuA4 and SAGA/SLIK [27], effectively also hyperlinking these two acetyltransferase complexes into Chromatin Central. Our attempts to TAP-tag Sc_Tra1p failed. However, Sc_Tra1p was co-purified when other Sc_NuA4 and also ASTRA subunits were sequentially tagged (Figure 2; also see Figure S2 in Additional data file 2 and Table S2 in Additional data file 1).

Notably, core-subunits of the histone deacetylase complex Sc_Set3C [10] were co-purified in sub-stoichiometric amounts with subunits of the Sc_Rpd3L complex (Table S2 in Additional data file 1). Sc_Set3C and Sc_Rpd3L complexes regulate overlapping target genes [33-35] and synthetic lethal screens have revealed genetic links between components of these complexes [36].

Altogether, the composition of individual complexes in Chromatin Central accords well with the published biochemical evidence. Furthermore, the sequential tagging approach revealed four novel subunits in three previously characterized complexes as well as three novel protein assemblies.

### Chromatin Central in *S. pombe*

**Figure 3:**
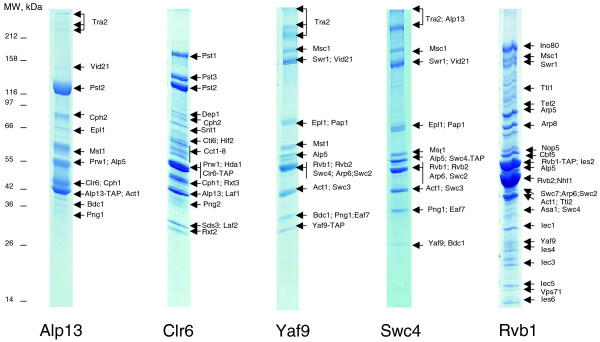
Representative Coomassie stained polyacrylamide gels of immunoaffinity isolations used for deciphering the Chromatin Central environment in *S. pombe*. These baits were selected for IP experiments as plausible proteomic hyperlinks. Bands, in which corresponding proteins were identified by mass spectrometry, are indicated with arrows for each lane. The full list of identified proteins is presented in Table S4 in Additional data file 1.

We next asked if the Chromatin Central environment is conserved between the distantly related fungi *S. cerevisiae *and *S. pombe*. In contrast to *S. cerevisiae*, no systematic biochemical isolation of protein complexes has yet been performed in *S. pombe*; however, complexes can be isolated with essentially the same TAP methodology with a similar success rate [7,37]. We exploited the architecture of Chromatin Central in *S. cerevisiae *to choose strategic baits for the work in *S. pombe*. The closest homologues of six *S. cerevisiae *hyperlinks (products of *CLR6*, *ALP13*, *YAF9*, *SWC4*, *RVB1*, *TRA1 *and *TRA2 *genes) were subjected to TAP tagging and immunoaffinity isolation, followed by mass spectrometric identification of corresponding interactors (Figure 3). For accuracy, we also isolated complexes associated with three more conserved subunits, encoded by *PNG2*, *SWC2* and *IES6*. Thus, the characterization of each complex relied upon at least two independent TAP purifications targeting different baits.

As in the *S. cerevisiae *experiments, the identified proteins, together with their A-indices, were combined into a master table (Tables S2 and S4 in Additional data file 1). We also compiled a list of 250 common background proteins for *S. pombe *in the same way as we did for *S. cerevisiae *(Table S1 in Additional data file 1). Interestingly, the average A-index of common background proteins was almost identical in both yeasts (1.3 and 1.4 in the fission and budding yeasts, respectively), and, therefore, we used the same conservative threshold of 1.75 to define stoichiometric interactors.

**Figure 4:**
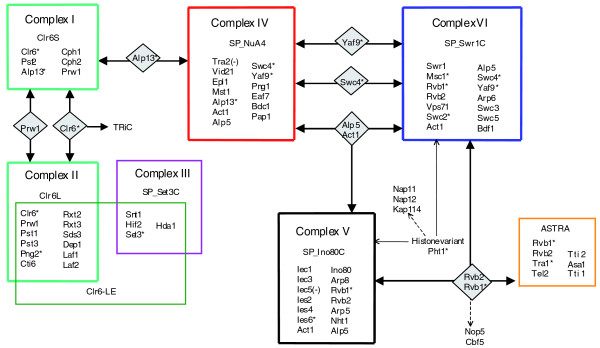
Chromatin Central proteomic environment in *S. pombe*. Individual protein complexes are boxed; color coding designates the protein complexes that are orthologous to the corresponding complexes in *S. cerevisiae *as shown in Figure 2. TAP-tagged subunits are designated by asterisks; minus (-) signs indicate that the IP experiment failed; proteomic hyperlinks are shown between the complexes in grey diamonds. Certain confident pair-wise interactors, discussed in the text, are designated with dashed arrows.

Chromatin Central shows a very similar architecture in both yeasts (Figures 2 and 4). To assess the similarities more closely, we focused on orthologues, recognized by overall sequence similarity (best hits in forward and reciprocal BLAST searches) and similar composition of structural domains (Tables 1, 2, 3, 4, 5). Altogether, in both Chromatin Central environments we identified 47 pairs of confident orthologues and six pairs with marginal confidence (Figure S3 in Additional data file 2) out of a total of 139 proteins. For other *S. cerevisiae *and *S. pombe *proteins, BLAST searches identified no clear orthologous pairs (Tables 1, 2, 3, 4), although some of them might be functional orthologues (such as Sc_Ume1p and Sp_Prw1p).

More than half the subunits of Sc_Rpd3S and Sc_Rpd3L (complexes I and II) are orthologous to the members of corresponding *S. pombe *complexes Sp_Clr6S and Sp_Clr6L; however, we reveal (Figure 4 and Table 2) further similarities than previously documented [38]. In addition to the previously reported subunits, we identified Sp_Cti6p, Sp_Rxt2p, Sp_Rxt3p, Sp_Dep1p and Sp_Pst3p. Our study also revealed that Sp_Clr6L, like Rpd3L in the budding yeast, is hyperlinked to the NuA4 histone acetyltransferase complex via an MRG-family protein, Sp_Alp13p.

Complex IV (Sp_NuA4) comprises orthologues of the 12 core members of the Sc_NuA4 complex, including its catalytic subunit Sp_Mst1p (ORF name SPAC637.12c) [39-41] (Table 1). Complexes V and VI include the closest homologues of the *S. cerevisiae *ATP-dependent helicases Sc_Swr1p and Sc_Ino80p (ORF names SPAC11E3.01c and SPAC29B12.01, respectively), together with 20 subunits orthologous to members of Sc_Swr1C and Sc_Ino80C (Table 3). The corresponding chromatin remodeling complexes in *S. cerevisiae *catalyze replacement of histone H2A with its variant Htz1p [29,42,43]. Complexes V and VI in the fission yeast both associate with Sp_Pht1p, which is the *S. pombe *orthologue of Sc_Htz1p (Table S2 in Additional data file 1). Therefore, it is likely that these *S. pombe *complexes (now called Sp_Swr1C and Sp_Ino80C) are also H2A.z chaperones.

Interestingly, while characterizing the composition of Sp_Ino80C, we identified a 17 kDa core subunit, whose gene has not been annotated as an ORF in the *S. pombe *genome (Figure S4 in Additional data file 2). The protein has no homologues within the *Saccharomyces *genus, yet possesses some remote similarity to a non-annotated genomic region in *Schizosaccharomyces japonicus*. We call this newly discovered *S. pombe *gene, *IEC5 *short for (*Ino Eighty Complex subunit 5 *[GenBank:FJ493251]).

Complex VI, ASTRA, is the same as the orthologous complex in *S. cerevisiae *except that the *S. pombe *genome encodes for two Tra1 homologues and only one, Tra1, is present in ASTRA (Table 4). The other, Tra2, is a subunit of Sp_NuA4 and presumably the *S. pombe *SAGA/SLIK complexes. In *S. cerevisiae*, the single Tra1 was found in all three complexes.

As we observed in *S. cerevisiae *for Sc_Rpd3L, some Sp_Set3C subunits co-purified in sub-stoichiometric amounts with Sp_Clr6L and vice versa, when Sp_Set3p was used as bait (Table S2 in Additional data file 1). Notably, the three subunits (Sp_Snt1p, Sp_Hif2p, and Sp_Set3p) isolated together with Clr6L are the orthologues of the three (Sc_Snt2, Sc_Sif2, and Sc_Set3) isolated with Rpd3L. However, in contrast to the Sc_Set3C complex, which consists of eight subunits, the Sp_Set3C complex contains only four proteins (Table 2).

In a few instances we identified proteins with domains that are not present in the corresponding orthologous complexes in the other yeast, including Sp_Msc1p (ORF name SPAC343.11c), which is a member of the Sp_Swr1C complex. The function of this protein is not known, although Ahmed *et al. *[44] suggested that Msc1 is involved in chromatin regulation and DNA damage response. Msc1 contains a remarkable composition of domains, including three PHD fingers [45], PLU-1 [46], zf-C5HC2, JmjC and JmjN [47]. It was recently shown that the Msc1 PHD fingers act as an E3 ubiquitin ligase [48], while in other proteins the JmjC domain mediates histone demethylation [49]. None of the Sc_Swr1C subunits possess these domains or appears to be remotely similar to Sp_Msc1 (Table S5 in Additional data file 2).

We identified nine hyperlinks within Chromatin Central in *S. pombe*, all of which are orthologues to corresponding proteins in the budding yeast. As our attempts to purify TRA2 failed (as they did in *S. cerevisiae*), it remains unclear if, similar to the budding yeast, this protein is also shared between Sp_NuA4 and an assembly orthologous to SAGA/SLIK [50].

### Independent validation of functional relationships within Set3C and Swr1C complexes

We independently validated some of the novel proteomics observations, namely the insights regarding Set3C and Swr1C, using quantitative genetic interaction data from *S. cerevisiae *[51] and *S. pombe *[52,53].

**Figure 5:**
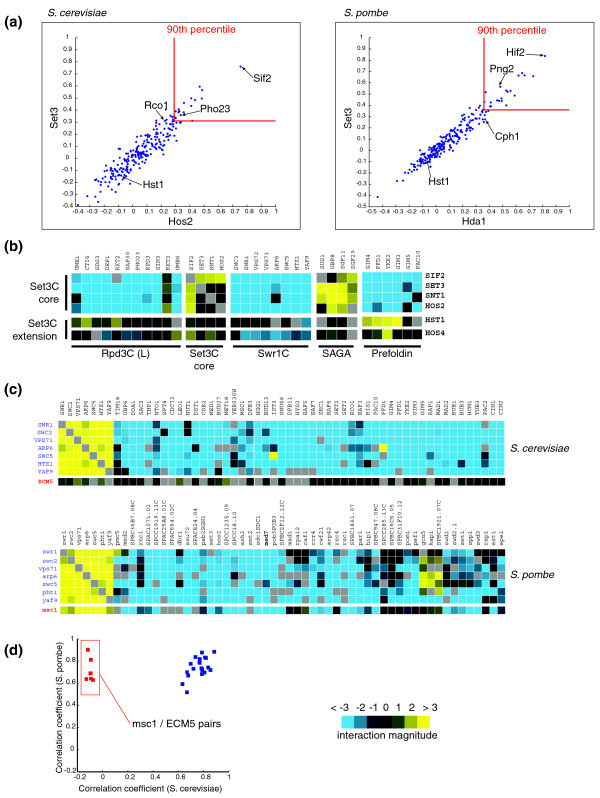
Genetic interactions support the proteomic observations. **(a) **Scatter plots of correlation coefficients between genetic profiles for the two Set3C subunits, Set3 and Hos2, in *S. cerevisiae *and *S. pombe *across 239 direct homologs between the two species. In both species Sif2 (Hif2) is highly correlated with both Set3 and Hos2, consistent with its being a subunit of the core Set3C, whilst Hst1 (part of the Sc_Set3C extension) is uncorrelated with either Set3 or Hos2. Pho23 (Png2), a subunit of Rpd3C(L), correlates better with Set3 and Hos2 than does Rco1 (Cph1), a subunit of Rpd3C(S). The 90th percentile of the data is indicated. **(b) **Subunits of the core Set3C and Set3C extension show different genetic interaction patterns. Shown are genetic interactions between Set3C core and extension subunits and Swr1C, SAGA and Prefoldin subunits. Color-coding of the interaction magnitude (shown in the key) is as follows: shades of cyan indicate synthetic sick/lethal (negative) interactions typically observed between genes acting on parallel pathways; shades of yellow represent suppressive (positive) interactions seen primarily between genes acting on the same pathway and within stable protein complexes. **(c) **Msc1 in *S. pombe *is a member of Swr1C, while its *S. cerevisiae *ortholog (Ecm5) is not. Genetic profiles of members of the complex in the two species are shown with Msc1 and Ecm5 profiles aligned at the bottom. Genetic pattern of Msc1 is very similar to the rest of the complex and positive genetic interactions with the other members indicate that it is a *bona fide *member of Swr1C in *S. pombe*. Color-coding is as for (b). **(d) **A scatter plot of pair-wise correlation coefficients of genetic profiles of members of Swr1C in *S. cerevisiae *and *S. pombe*. Consistent with (c), data-points corresponding to pairs containing Msc1 or Ecm5 form an outlier group and are strongly correlated in *S. pombe*, but not in *S. cerevisiae*.

Our proteomic data suggest that Set3C contains a conserved core complex of four proteins (Set3, Hos2, Snt1, and Sif2) and physically interacts with Rpd3L in both *S. cerevisiae *and *S. pombe*. To validate these findings, we compared the correlation coefficients of genetic profiles of the two Set3C core components in both yeasts (Sc_Set3 and Sc_Hos2; Sp_Set3 and Sp_Hda1) against genetic profiles of a set of 239 direct homologs in the two species. As expected, the correlation between the two core members (Set3 and Hos2) and Sif2, is well beyond the 90th percentile (Figure 5a), suggesting they act in concert. In contrast Hst1, also a subunit of Sc_Set3C, shows a much lower correlation, which is consistent with its absence from the core complex (as was previously shown for Sc_Hst1 [10]) or not being a part of the complex at all (Sp_Hst1). Furthermore, correlation of the Set3C core with the Sc_Rpd3L subunit Sp_Pho23 (Sp_Png2) is also high in both yeasts and higher than the correlation with one of the Rpd3S subunits (Rco1, Sp_Cph1). The functional division within Sc_Set3C becomes even more obvious when examining individual interactions of Set3C core and extension subunits. Members of the Set3C core display stronger positive genetic interactions with each other, compared to the Set3C extension subunits, and their genetic interaction patterns differ from patterns of Swr1C, SAGA and Prefoldin members (Figure 5b). Taken together, these data provide genetic evidence that Sc_Set3C encompasses two functional modules, one of which (Set3C core) interacts closely with Rpd3L. This functional evidence corroborates our proteomic mapping data, suggesting that the Set3C complex in *S. pombe *is only represented by core subunits, while the orthologous complex in *S. cerevisiae *has an extension of four extra subunits. In both yeasts, the core Set3C interacts with Rpd3L to form a distinct module referred to as Rpd3LE.

In *S. pombe *another complex, Swr1C, contains a newly identified subunit, Msc1. Its closest homolog in Sc_Ecm5 is not a part of Swr1C in budding yeast. To independently validate this finding, we examined and compared individual genetic interactions of seven of the Swr1C subunits in both yeasts with the genetic patterns of Sp_Msc1 (Sc_Ecm5). Consistent with our proteomic data, Sp_Msc1, unlike Sc_Ecm5, shows strong positive genetic interactions and a very similar pattern to the other members of the complex (Figure 5c). Hence, pairs of genetic profiles containing Sc_Ecm5/Sp_Msc1 and other members of Swr1C show weak correlation in *S. cerevisiae*, but strong correlation in *S. pombe *(Figure 5d). Taken together, these genetic data confirm our proteomic mapping observations.

## Discussion

By navigating a complex proteomic environment in two divergent yeasts with high accuracy, we obtained a new level of precise insight into the comparative proteome and also extracted several new and subtle discoveries.

### Comparative profile of a proteomic environment

The overall architecture of Chromatin Central is the same in the two yeasts; however, there is a surprising amount of variation in their subunit composition. For both yeasts, Chromatin Central is based on the same eight complexes, encompassing 53 orthologous protein pairs plus a further 33 proteins that appear to be species specific (23 in *S. cerevisiae*, 10 in *S. pombe*). Of these 33, three pairs have a very similar composition of functional domains (Table S5 in Additional data file 2). Hence, a very similar architecture is sustained by a scaffold based on about two-thirds of all the proteins involved, whereas the remaining one-third appears to be less constrained.

Analyses of domain composition (Table S5 in Additional data file 2) revealed that many subunits (19 in *S. cerevisiae *and 21 in *S. pombe*) possess bromo-, chromo-, SANT or PHD finger domains, which can bind either methylated or acetylated histones or other chromatin determinants [30,54-57], thus potentially targeting their complexes to specific regions of chromatin. Along with the seven enzymes in Chromatin Central, these putative targeting subunits are the most highly conserved subunits between the two yeasts. For example, Sc_NuA4 and Sp_NuA4 complexes retain all four targeting factors: the PHD fingers of Sc_Yng2/Sp_Png2; the bromodomains of Sc_Bdf1/Sp_Bdc1 and the chromodomains of Sc_Eaf3/Sp_Alp13 and Sc_Esa1/Sp_Mst1. Similarly, the different PHD finger subunits of Rpd3S and Rpd3L (Sc_Rco1 and Sp_Cph2 in Rpd3S/Clr6S; Sc_Cti6 and Sp_Cti6 as well as Sc_Pho23 and Sp_Png2 in Rpd3L/Clr6L), which appear to direct these complexes to differentially methylated nucleosomes [58], are conserved. Hence, almost half of the conserved scaffold of Chromatin Central is based on proteins that convey the functions of the environment, that is, the reading and writing of the histone code.

### Comparative proteomics and proteomic hyperlinks

In contrast to the conserved scaffold of Chromatin Central, the proteomic hyperlinks appear to be less constrained. We define proteomic hyperlinks, which are notable features of proteomic environments, as proteins found as stoichiometric subunits of more than one scaffold complex. Hyperlinks do not physically connect complexes; rather, they could exist for one of the following three reasons. First, hyperlinks might reflect a common ancestry for two complexes. In Chromatin Central, it is possible that Ino80C and Swr1C are examples of complexes that share a common evolutionary origin because they not only share four subunits, but also share a similar function related to the histone variant H2A.z [43,59]. Second, hyperlinks may play a functional role to co-ordinate two complexes. If the hyperlink receives a signal via a post-translational modification, then two complexes should receive the signal at the same time and, hence, be co-regulated. Conversely, if a hyperlink recognizes an epitope or target, then both complexes will be coordinately recruited. In Chromatin Central, the Rpd3S/NuA4 hyperlink Eaf3 plays a role in coordinating these complexes [60]. Third, the hyperlink may be a common tool recruited to the complex. In Chromatin Central, the Rvb1/Rvb2 heterodimeric helicase is a good example to consider. It is present in three complexes, Swr1C, Ino80C and ASTRA, presumably because each requires a helicase for action in chromatin.

Altogether, we distinguished 21 proteomics hyperlinks in both Chromatin Central environments, 12 in *S. cerevisiae *and 9 in *S. pombe *(Figures 2 and 4; Table S6 in Additional data file 1). These proteins display a variety of physicochemical properties and domains (Table S5 in Additional data file 2 and Table S6 in Additional data file 1). For instance, in *S. cerevisiae *four hyperlink proteins are enzymes: Sc_Rvb1p and Sc_Rvb2p are DNA helicases, Sc_ Rpd3p is a histone deacetylase, and Sc_Bdf1p is a protein kinase. Sc_Act1p and Sc_Arp4p are cytoskeleton and structural proteins. Sc_Tra1p belongs to a protein kinase family (although its catalytic activity has been questioned [61]), whereas no enzymatic activity has been reported for the other three proteins, Sc_Eaf3p, Sc_Yaf9p, Sc_Swc4p. Thus, the hyperlinks display diverse functional roles. However, they are all members of highly conserved protein families with clear homologues even in vertebrates. Also, half of the *S. cerevisiae *hyperlinks (6 out of 12) are essential, whereas only 3 essential genes were additionally found among the other 73 members of the environment.

Of the twelve hyperlinks in *S. cerevisiae *Chromatin Central, three are not conserved between the two yeasts. In two out of these three cases, the lost hyperlink is due to gene duplication or deletion. For Rpd3S and Rpd3L, Sin3 is a hyperlink in *S. cerevisiae*, but in *S. pombe*, both Clr6S and Clr6L have a dedicated Sin3 homologue. In fact, *S. pombe *has three Sin3 paralogues, with Sp_Pst2p being exclusively found in Clr6S whereas the other two homologues, Sp_Pst1p and Sp_Pst3p, are exclusively found in Clr6L. For NuA4, SAGA/SLIK and ASTRA, Tra1 is a hyperlink in *S. cerevisiae*, but in *S. pombe*, the *tra1 *gene is duplicated with one paralogue present in ASTRA and another in NuA4 and, presumably, SAGA/SLIK. We have previously noted the same phenomenon regarding a lost hyperlink. In *S. cerevisiae*, Swd2 hyperlinks Set1C and CPF; however, in *S. pombe *these two complexes each have a dedicated Swd2 paralogue, Swd2.1 and Swd 2.2. Notably, deletion of the gene encoding Swd2.1 did not provoke Swd2.2 to occupy the missing position in Sp_Set1C [7]. In the third case, Bdf1 is a hyperlink between NuA4 and Swr1C in *S. cerevisiae*, but in *S. pombe*, it is replaced by two non-orthologous proteins with a similar composition of domains (Tables 1 and 3; Table S5 in Additional data file 2).

**Figure 6:**
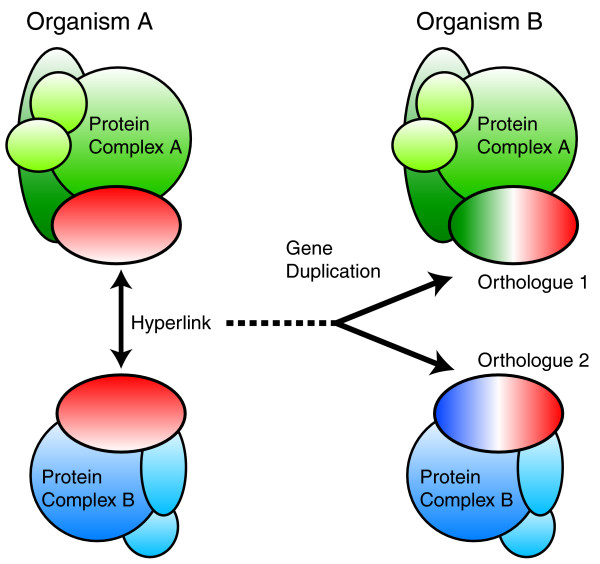
Several divergent proteomic hyperlinks between *S. cerevisiae *and *S. pombe *are due to gene duplications. Hence, hyperlinks provide a mechanism for the fixation of gene duplications.

Although we have documented only a few examples, this early concordance between hyperlinks and gene duplications/deletions is notable and indicates that a gene duplication in evolution may be especially advantageous when it is a proteomic hyperlink. Gene duplication permits the diversification of the encoded protein. However, unless all genes encoding interacting proteins, particularly members of the corresponding protein complex, are also duplicated, diversification of the duplicated protein will be constrained by the existing spectrum of protein-protein interactions [62]. If the duplicated gene encodes a hyperlink, then diversification of the duplicated protein will be less constrained because the duplications can associate and specialize with different complexes (Figure 6). Therefore, the gene duplication of a hyperlink may be more successful than other gene duplications. From a technical point of view, we suggest that the tagging of hyperlinks as an entry point for mapping proteomic environments will be a rewarding focus for mapping and understanding proteomes.

### New complexes in Chromatin Central

Despite the fact that this work was based on one of the most intensely studied proteomic environments to date, we documented four new subunits in the six known complexes (described above in Results). We also found three new complexes, including two more containing the histone deacetylase Rpd3, to add to the recently described Rpd3S and Rpd3L complexes [24,25].

**Figure 7:**
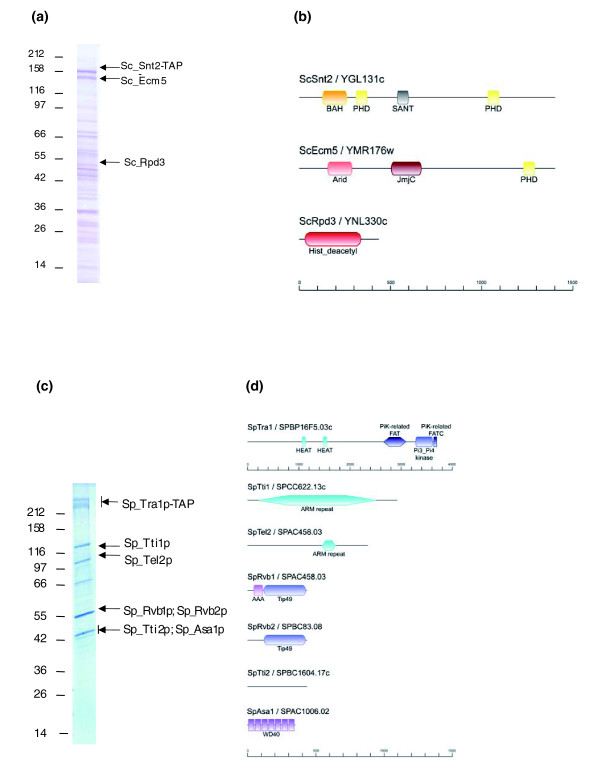
New protein complexes in Chromatin Central. **(a) **Immuno-isolation of the Sc_Snt2C complex and **(b) **domain structure of its subunits. **(c) **Immuno-isolation of the Sp_ASTRA complex and **(d) **domain structure of its subunits. In (a, c), only the relevant protein bands are annotated. The full list of identified proteins is provided in Table S1 in Additional data file 1.

We found the three-member Sc_Snt2 complex in an Rpd3-TAP pull down. Subsequently, we validated the complex by tagging Snt2 (Figure 7a). Sc_Snt2p and the other subunit, Sc_Ecm5p, contain several domains involved in chromatin regulation (Figure 7b). Sc_Snt2p (YGL131C) is one of 18 SANT domain-containing proteins in budding yeast and the sixth identified in Chromatin Central (Table S5 in Additional data file 2). Due to its JmjC domain, Sc_Ecm5p is a putative histone demethylase. Its PHD fingers have been reported to bind methylated H3K36 [58].

We did not find a complex similar to Sc_Snt2C in *S. pombe*. Previously, we showed that the fission yeast orthologue of Sc_Snt2p is also found in a complex with the JmjC domain proteins Sp_Lid2p and Sp_Jmj3p [7,17]. However, these two complexes, Sc_Snt2C and Sp_Lid2C, are very different. From our IPs and Coomassie gels, we note that the Sc_Snt2C is much less abundant than either Rpd3S or Rpd3L.

The second new Rpd3-containing complex, Rpd3-LE, also contains a minor fraction of cellular Rpd3. Rpd3-LE is an extension of the Rpd3L complex, which includes the core of the Set3 complex. We have four reasons to be confident of this assignment. First, we reciprocally observed the relationship between Rpd3L and core Set3C in both yeasts. Second, in *S. cerevisiae *we observed only the core proteins of Set3C, but not the full eight-membered Set3C, associated with Rpd3L. This accords with our previous dissection of Sc_Set3C, which showed that the complex has a core of these four proteins [10]. Third, Sp_Set3C consists of only the four core proteins found in Sc_Set3C and does not include homologues of Sc_Hst1p or Sc_Hos4p. It also does not include a homologue of the cyclophilin Sc_Cph1p, which is required to fine-tune the sporulation-specific activity of Sc_Set3C [10,63]. Fourth, genetic interaction data clearly support the division of Sc_Set3C into core and extension components, as well as the interaction of the core with Rpd3L.

Taken together, these data indicate that Set3C has two versions, one that is small, common to both yeasts and interacts with Rpd3L, and the another that is larger and specific to *S. cerevisiae*. Sc_Set3C functions during vegetative growth to regulate several inducible genes and to co-operate with Rpd3 [33-35]. It has also been shown to be a negative regulator during meiosis [10]. Partly because transcriptional control of meiosis appears to be poorly conserved [64], we suggest that the vegetative functions in *S. cerevisiae *entirely relate to the smaller complex.

We also discovered the ASTRA complex, which is composed of orthologous subunits in both yeasts and contains a Tra1 member of the ATM-like kinase family (Figures 2, 4 and 7C,D; Table 4). ASTRA includes the ATP-dependent DNA helicase Rvb1/2 heterodimer, the telomere binding protein Tel2p together with two uncharacterized Tel2-interacting proteins (Tti1p and Tti2p), and a WD-repeat containing protein (Asa1p) of unknown function in both yeasts.

Protein assemblies similar to ASTRA have not been identified in other organisms. However, five subunits have clear human homologues so the complex may be widespread. Furthermore, Sc_Tel2p, as well as the *C. elegans *(CLK-2 [65]) and human (TELO [66]) orthologues are required for telomere length regulation. Sc_Tel2p also influences telomere position effect and interacts directly with double-stranded telomeric DNA [67,68]. Recently, Hayashi *et al. *[69] suggested that a Tel2-Tti1 heterodimer recognizes a common domain of phosphatidyl inositol 3-kinase related kinases (PIKK) in the fission yeast and is a component of multiple PIKK assemblies, which corroborates our findings in *S. cerevisiae *(Table S2 in Additional data file 1). Hence, we suggest that ASTRA is the Tra-specific Tel2/CLK-2/TELO complex involved in telomeric chromatin regulation and will be found in many eukaryotes.

The stringency of our protein selection criteria may have excluded some lowly abundant interactors, as well as proteins only interacting with a particular subunit of the complex, rather than with the entire complex cores. This might include several interesting possible further connections to Chromatin Central (Tables S2, S3 and S4 in Additional data file 1). For example, we observed several subunits of the SWI-SNF global transcription activator complex [70-72], RSC chromatin remodeling complex [72,73], Regulator of Nucleolar silencing and Telophase exit (RENT) [74,75], Anaphase Promoting Complex (APC) [37], cytoplasm to nucleus signaling complexes TORC1 and TORC2 [69,76], the proteasome, DNA replication licensing complex [77], the CCR4/NOT transcription complex [78] and various transcription factors.

The histone deacetylases Rpd3p and Clr6p pulled down the complete TRiC chaperonin complex [79] in *S. cerevisiae *and *S. pombe*, respectively (Figures 2 and 4; Tables S3 and S4 in Additional data file 1). Similarly, TRiC was co-purified with the Hos2 deacetylase complex Set3C in *S. cerevisiae *[5]. Hence, we suggest that TriC chaperonin activity is specifically related to type I histone deacetylases.

## Conclusion

Comparative proteomics remains undeveloped because the generation of reliable proteomic data remains challenging. A variety of candidate approaches using synthetic expression libraries, bioinformatics, or high throughput methodologies have been applied to tackle the challenge. The best datasets have been acquired by affinity purification approaches based on authentic expression levels. Strategies to apply this conclusion at a genome scale have been developed for yeast [11,12] and are being developed for mammalian systems [80]. Based on the conclusions drawn here, we suggest that comparative proteomics will become a valuable complement for proteomic mapping because it presents alternative ways to validate data. Proteins interacting with Set3 illustrate this point well. In *S. cerevisiae*, the interaction between Rpd3L and the core of Set3C is sub-stoichiometric and obscured by the existence of alternative Rpd3 and Set3 complexes. However, our reciprocal identification of the Rps3-LE complex in both yeasts secures the observation, which was also supported by genetic interaction studies in both yeasts. Comparative proteomics can also guide the investigation of new proteomes. In particular, projecting yeast data onto mammalian proteomes has relevance for medicine. Although the available datasets are incomplete, protein assemblies that are, apparently, orthologous to the yeast complexes NuA4, Swr1C, INO80C and Set3C, along with Rpd3 interactors, have been partially characterized (Figures S5 and S7 in Additional data file 2) [81-92] and suggest a highly conserved proteomic environment. Potentially, the orthologous complexes in humans are hyperlinked into a similar scaffold architecture, although different protein orthologues are recruited as hyperlinks. Because of multiple gene duplications, human assemblies are more complicated (such as hHDACs). However, comparative proteomics can guide the search for missing human subunits or even protein complexes, like the ASTRA orthologue.

Our intense focus on a section of the *S. cerevisiae *proteome also revealed new details. These further gains in proteomic accuracy are partly due to recent performance improvements delivered by LC-MS/MS. The improved capacity for protein identification above background and noise in complex mixtures permits a greater ability to distinguish sub-stoichiometric interactors from background. This greater depth has implications for biochemical practice in two ways. First, the reliance on candidate approaches to map the proteome, such as two-hybrid or bioinformatics, has been lessened because affinity chromatography and mass spectrometry can be used to deliver reliable sub-stoichiometric data, in addition to the well established capacity to document stoichiometric interactions. Second, classic biochemistry, which includes tagging approaches like the TAP method, delivered highly purified fractions based on multiple purification steps. However, weak interactions were inevitably lost during multiple steps of purification. Now, a new logic is emerging based on minimizing the biochemical procedure so that more weak and sub-stoichiometric interactions are preserved. Although these less purified preparations will have increased background, mass spectrometry can now identify large numbers of proteins in complex mixtures. Its combination with protein quantification and bioinformatics should largely eliminate background proteins, thus opening new paths to map proteomes accurately at greater depth. Given greater accuracy, comparative proteomics will become a leading source of insight into eukaryotic cellular and developmental mechanics.

## Materials and methods

### Epitope tagging of genes and isolation of protein complexes

Transformations for both yeasts were performed as described [7,9]. Genes of interest were tagged by in-frame fusion of the ORFs with a PCR generated targeting cassette encoding the TAP-tag and a selectable marker. Correct cassette integrations were confirmed by PCR and Western blot analysis. Two *S. cerevisiae *strains with TAP-tagged genes YGR099W and YJR136C were obtained from Euroscarf (Frankfurt am Main, Germany). Breaking and extraction of yeast cells was performed as described [7] with modifications [10]. Purified proteins were concentrated according to Wessel and Fluegge [93] and loaded onto one-dimensional gradient (6-18%) polyacrylamide gels.

### Protein separation and in-gel digestion

Protein bands were visualized by staining with Coomassie. Full lanes were cut into approximately 30-40 slices; to enhance the detection dynamic range, visible bands were always sliced separately. Excised gel plugs were cut into approximately 1 mm × 1 mm × 1 mm cubes and in-gel digested with sequencing grade modified porcine trypsin (catalogue number V5111, Promega, Mannheim, Germany) as described in [94]. Then, 1 μl aliquots were withdrawn directly from in-gel digests for the protein identification by MALDI peptide mapping. The rest of the peptide material was extracted from the gel pieces with 5% formic acid and acetonitrile and recovered peptides dried down in a vacuum centrifuge.

### Protein identification by MALDI peptide mass mapping

Where specified, 1 μl aliquots of in-gel digests were analyzed on a REFLEX IV mass spectrometer (Bruker Daltonics, Bremen, Germany) using AnchorChip probes (Bruker Daltonics) as described in [95,96]. Peaks were manually selected and their *m/z *searched against MSDB protein database of *S. cerevisiae *or *S. pombe *species using MASCOT 2.0 software (Matrix Science Ltd, London, UK), installed on a local two CPU server. Mass tolerance was set to 50 ppm; variable modifications: oxidized methionines; one misscleavage per tryptic peptide sequence was allowed. Spectra were calibrated externally using *m/z *of known abundant trypsin autolysis products as references. Protein hits whose MOWSE score exceed the value of 51 (the threshold confidence score suggested by MASCOT for *p *< 0.05 and the corresponding species-specific database) were considered significant, but were only accepted upon further manual inspection, which made sure that the *m/z *of all major peaks in the spectrum matched the masses of peptides from the corresponding protein sequences or known tryptic autolysis products.

### Protein identification by LC-MS/MS

Dried peptide extracts were re-dissolved in 20 μl of 0.05% (v/v) trifluoroacetic acid and 4 μl were injected using a FAMOS autosampler into a nanoLC-MS/MS Ultimate system (Dionex, Amstersdam, The Netherlands) interfaced on-line to a linear ion trap LTQ mass spectrometer (Thermo Fisher Scientific, San Jose, CA, USA). The mobile phase was 95:5 H_2_O:acetonitrile (v/v) with 0.1% formic acid (solvent A) and 20:80 H_2_O:acetonitrile (v/v) with 0.1% formic acid (solvent B, *Lichrosolv *grade). Peptides were first loaded onto a trapping microcolumn C18 PepMAP100 (1 mm × 300 mm ID, 5 mm, Dionex) in 0.05% trifluoroacetic acid at a flow rate of 20 ml/minute. After 4 minutes they were back-flush eluted and separated on a nanocolumn C18 PepMAP100 (15 cm × 75 μm ID, 3 μm, Dionex, Sunnyville, CA, USA) at a flow rate of 200 nl/minute in the mobile phase gradient: from 5-20% of solvent B in 20 minutes, 20-50% B in 16 minutes, 50-100% B in 5 minutes, 100% B during 10 minutes, and back to 5% B in 5 minutes; %B refers to the solvent B content (v/v) in A+B mixture. Peptides were infused into the mass spectrometer via a dynamic nanospray probe (Thermo Fisher Scientific) and analyzed in positive mode. Uncoated needles Silicatip, 20 μm ID, 10 μm tip (New Objective, Woburn, MA, USA) were used with a spray voltage of 1.8 kV, and the capillary transfer temperature was set to 200°C. In a typical data-dependent acquisition cycle controlled by Xcalibur 1.4 software (Thermo Fisher Scientific), the four most abundant precursor ions detected in the full MS survey scan (*m/z *range of 350-1,500) were isolated within a 4.0 amu window and fragmented. MS/MS fragmentation was triggered by a minimum signal intensity threshold of 500 counts and carried out at the normalized collision energy of 35%. Spectra were acquired under automatic gain control in one microscan for survey spectra and in three microscans for MS/MS spectra with a maximal ion injection time of 100 ms per each microscan. *M/z *of the fragmented precursors were then dynamically excluded for another 60 s. No precompiled exclusion lists were applied.

MS/MS spectra were exported as dta (text format) files using BioWorks 3.1 software (Thermo Fisher Scientific) under the following settings: peptide mass range, 500-3,500 Da; minimal total ion intensity threshold, 1,000; minimal number of fragment ions, 15; precursor mass tolerance, 1.4 amu; group scan, 1; minimum group count, 1.

### Processing of MS/MS spectra and database searches

Dta files were merged into a single MASCOT generic format (mgf) file and searched against a database of *S. cerevisiae *or *S. pombe *proteins using MASCOT v.2.2 installed on a local two CPU server. Tolerance for precursor and fragment masses was 2.0 and 0.5 Da, respectively; instrument profile, ESI-Trap; variable modification, oxidation (methionine); allowed number of miscleavages, 1; peptide ion score cut-off, 15.

Hits were considered confident if two or more MS/MS spectra matched the database sequences and their peptide ion scores exceeded the value of 31 (the threshold score suggested by MASCOT for confident matching of a single peptide sequence at *p *< 0.05). For each identified protein, the number of matched peptides and of MS/MS spectra were exported from MASCOT output to Excel spreadsheets using a script developed in-house, which further created a non-redundant list of protein hits detected in all analyzed bands of the same IP experiment. If the same protein was sequenced in several bands, only the analysis that produced the highest number of matched peptides and spectra was reported.

### Protein identification in the *S. pombe *genome database

Where specified, recovered tryptic peptides were sequenced *de novo *by nanoelectrospray tandem mass spectrometry on a QSTAR Pulsar *i *quadrupole time-of-flight mass spectrometer (MDS Sciex, Concord, Canada) as described [97]. MS/MS spectra were interpreted manually using BioAnalyst QS v.1.1 software and candidate sequences searched against the genomic sequence of *S. pombe *by the *tblastn *program at the NCBI BLAST server. The search found, with several matched peptides, a non-annotated segment at chromosome 1. Subsequently, the full length sequence of the gene was produced by 5'-RACE and determined at the DNA sequencing facility in MPI CBG, Dresden.

### Bioinformatic identification of orthologous genes

Protein sequence database searches were carried out using a stand-alone version of NCBI-BLAST and the PSI-BLAST (position-specific iterated BLAST) interface at the NCBI [98]. To identify orthologous genes in *S. cerevisiae *and *S. pombe*, we performed automated BLAST searches using sequences of all subunits of all identified complexes. Potential orthologues were further evaluated by reciprocal BLAST searches using all hits whose *E*-values were less than 10-fold higher than the *E*-value of the best hit. Best hits in reciprocal searches were regarded as orthologues. If no reciprocal best hit pair was identified, PSI-BLAST searches were carried out against all fungal sequences in the non-redundant protein database. *E*-values and PSI-BLAST iterations for highly divergent orthologues are shown in Figure S3 in Additional data file 2. Multiple sequence alignments also shown in Figure S3 in Additional data file 2 were done manually by using pair-wise alignments produced by BLAST as a template. Residues that were conserved in at least 75% of sequences are highlighted. Identification of protein sequence domains was carried out using a stand-alone version of the InterproScan software [99] against the Superfamily and HMM-Pfam databases using default settings.

### Genetic interactions

Quantitative genetic interaction profiles in *S. cerevisiae *and *S. pombe *were generated as described [51,100]. Pearson correlation coefficients were calculated between all possible pairs of genetic profiles for an overlapping set of genes in both species and the data corresponding to Set3 and Hos2 (Hda1) are presented as scatter plots in Figure 5.

## Abbreviations

A-index: Abundance index; IP: immunoaffinity purification; LC-MS/MS: liquid chromatography tandem mass spectrometry; MALDI MS: matrix-assisted laser desorption/ionization mass spectrometry; ORF: open reading frame; TAP: tandem affinity purification.

## Authors' contributions

AR, DS, LB and CS performed gene tagging and IP experiments. Anna S and HT analyzed the isolated proteins by mass spectrometry. Anna S processed the identification data and determined Chromatin Central architecture in both yeasts. BH identified pairs of orthologous genes and determined functional domains in Chromatin Central proteins. AR and NK performed the genetic interaction experiments. Andrej S and AFS conceived the study, and together with other co-authors interpreted the results and wrote the manuscript. All authors have read and approved the manuscript.

## Additional data files

Additional data file 1 contains five worksheets. Table S1 lists common background proteins observed in TAP experiments in *S. cerevisiae *and *S. pombe*. Table S2 presents full lists of proteins identified in all immunoprecipitation experiments in both yeasts. Tables S3 and S4 are master tables of identified proteins used for compiling Chromatin Central in *S. cerevisiae *and *S. pombe*, respectively. Table S6 lists physical properties and bioinformatic annotations of proteomics hyperlinks. Additional data file 2 contains five figures and two tables in pdf format. Figure S1 plots A-indices of six protein standards versus corresponding protein loadings. Figure S2 presents gel images of immunoprecipitation experiments used for compiling Chromatin Central in *S. cerevisiae*. Figure S3 presents multiple sequence alignments for several members of Chromatin Central in *S. cerevisiae*, whose similarity to corresponding *S. pombe *proteins was marginal. Figure S4 presents mass spectrometric identification of the novel 17 kDa protein in *S. pombe*, its full-length amino acid sequence and its alignment with the corresponding region of the genome. Figure S5 presents a plausible molecular architecture of the human Chromatin Central (partly supported by already published evidence). Table S5 lists domain composition of orthologous complexes within Chromatin Central in both yeasts. Table S7 lists plausible members of human Chromatin Central, considering their homology to corresponding proteins in both yeast proteomic environments and published evidences.

## Supplementary Material

Additional data file 1 Table S1: common background proteins observed in TAP experiments in *S. cerevisiae *and *S. pombe*. Table S2: full lists of proteins identified in all immunoprecipitation experiments in both yeasts. Tables S3: master table of identified proteins used for compiling Chromatin Central in *S. *cerevisiae. Table S4 master table of identified proteins used for compiling Chromatin Central in *S. pombe*. Table S6: physical properties and bioinformatic annotations of proteomics hyperlinks. Click here for file

Additional data file 2 Figure S1: A-indices of six protein standards versus corresponding protein loadings. Figure S2: gel images of immunoprecipitation experiments used for compiling Chromatin Central in *S. cerevisiae*. Figure S3: multiple sequence alignments for several members of Chromatin Central in *S. cerevisiae*, whose similarity to corresponding *S. pombe *proteins was marginal. Figure S4: mass spectrometric identification of the novel 17 kDa protein in *S. pombe*, its full-length amino acid sequence and its alignment with the corresponding region of the genome. Figure S5: a plausible molecular architecture of the human Chromatin Central (partly supported by already published evidence). Table S5: domain composition of orthologous complexes within Chromatin Central in both yeasts. Table S7: plausible members of human Chromatin Central, considering their homology to corresponding proteins in both yeast proteomic environments and other published evidences. Click here for file
