# Supplementary material for: Chromatin Central: towards the comparative proteome by accurate mapping of the yeast proteomic environment
Source: Genome Biol. 2008 Nov 28;9(11):R167. doi: 10.1186/gb-2008-9-11-r167 (PMC2614481; doi:10.1186/gb-2008-9-11-r167)
Supplement: Additional data file 2 — Figure S1: A-indices of six protein standards versus corresponding protein loadings. Figure S2: gel images of immunoprecipitation experiments used for compiling Chromatin Central in S. cerevisiae. Figure S3: multiple sequence alignments for several members of Chromatin Central in S. cerevisiae, whose similarity to corresponding S. pombe proteins was marginal. Figure S4: mass spectrometric identification of the novel 17 kDa protein in S. pombe, its full-length amino acid sequence and its alignment with the corresponding region of the genome. Figure S5: a plausible molecular architecture of the human Chromatin Central (partly supported by already published evidence). Table S5: domain composition of orthologous complexes within Chromatin Central in both yeasts. Table S7: plausible members of human Chromatin Central, considering their homology to corresponding proteins in both yeast proteomic environments and other published evidences. [file gb-2008-9-11-r167-S2.pdf]

Figure S1

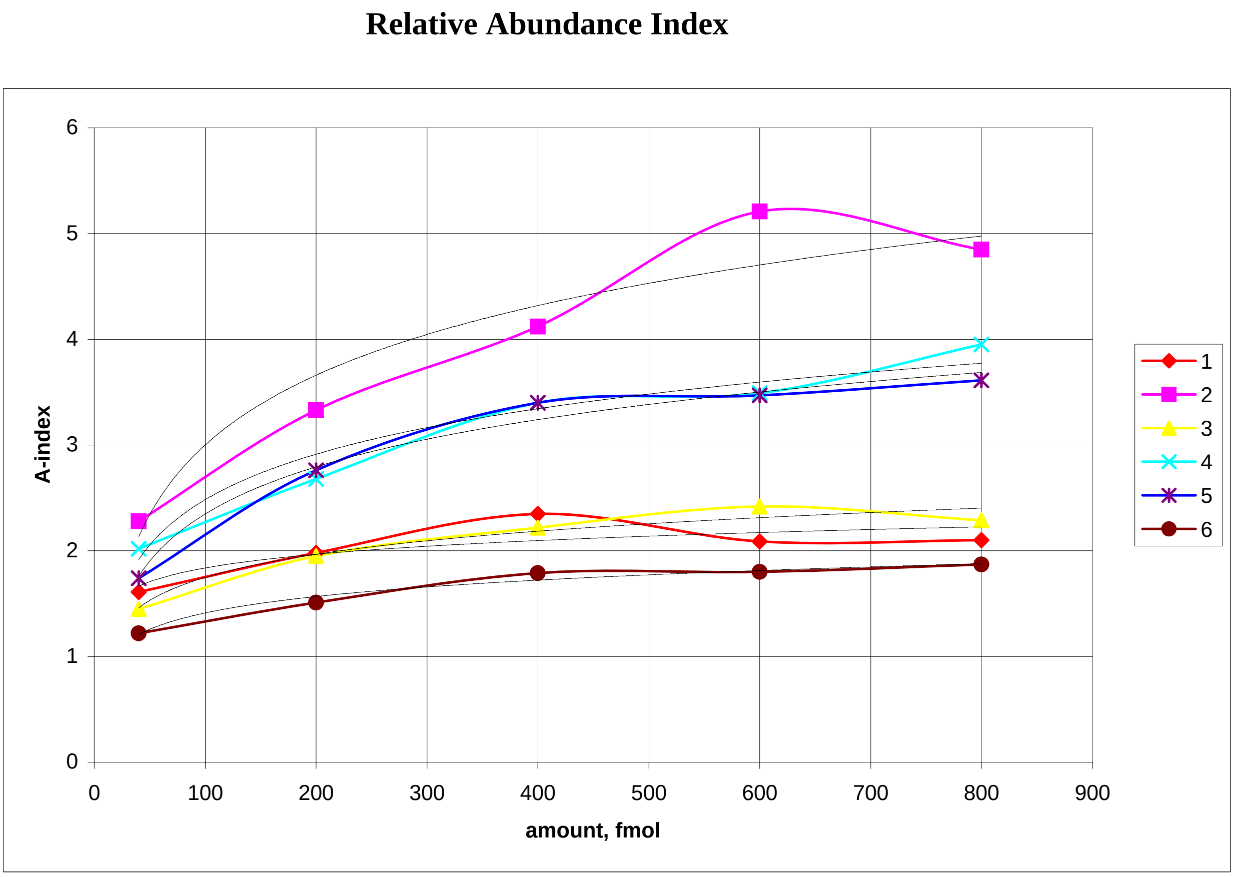

Relative Abundance Index (A-index) was calculated as a ratio between the total number of MS/MS spectra and the number of unique peptides matched to the protein sequence.

$$\text{A-index} = \frac{\text{N of spectra}}{\text{N peptides}}$$

In other words, A-index indicates the average number of MS/MS events triggered by the precursor ion matched to a given protein sequence.

The figure shows how A-indexes calculated for various proteins under fixed LC-MS/MS settings depends on the protein loading. The standard equimolar mixture of six pre-digest proteins (purchased from LC Packings, CA) was diluted to the final concentration of 10, 50, 100, 150 and 200fmol/µl and four microliters were injected and analyzed on LTQ instrument as described in Materials and methods section. Each experiment was repeated three times and the results were averaged. Line

Trendlines (logarithmic scale) are:
1 - Cytochrome C (MW 11kDa, pI 9.52)
$y = 0.187Ln(x) + 0.9768; R^2 = 0.6885$
2 - Lysozyme (14kDa, pI 9.18)
$y = 0.9505Ln(x) - 1.3763; R^2 = 0.9209$
3. - Alcohol dehydrogenase (37kDa, pI 6.21)
$y = 0.315Ln(x) + 0.2984; R^2 = 0.9562$
4 – Albumin (70kDa, pI 5.82)

y = 0.6203Ln(x) - 0.3732; R^2 = 0.9519
5 – Transferrin (78kDa, pI 6.75)
y = 0.6436Ln(x) - 0.616; R^2 = 0.9859
6 - Beta-galactosidase (116kDa, pI5.28)
y = 0.2239Ln(x) + 0.3816; R^2 = 0.972

Figure S2

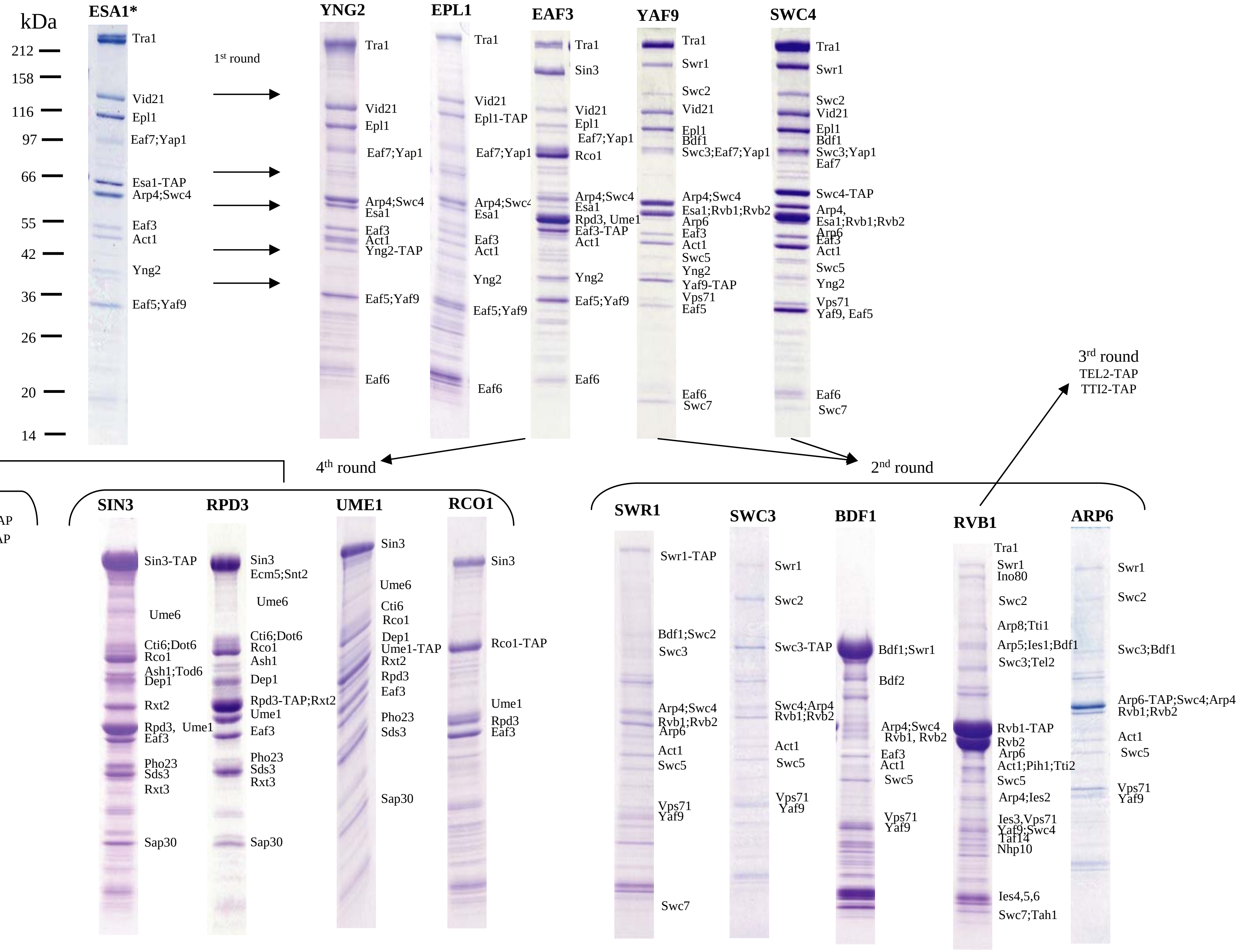
ESA1*
kDa
212
158
116
97
66
55
42
36
26
20
14
Tra1
Vid21
Epl1
Eaf7;Yap1
Esa1-TAP
Arp4;Swc4
Eaf3
Act1
Yng2
Eaf5;Yaf9
1st round
YNG2
Eaf7;Yap1
Arp4;Swc4
Esa1
Yng2-TAP
Eaf5;Yaf9
Eaf6
EPL1
Epl1-TAP
EAF3
Sin3
Rco1
Rpd3, Ume1
Eaf3-TAP
YAF9
Swr1
Swc2
Bdf1
Swc3;Eaf7;Yap1
Esa1;Rvb1;Rvb2
Arp6
Swc5
Yaf9-TAP
Vps71
Eaf5
Swc7
SWC4
Swc3;Yap1
Eaf7
Swc4-TAP
Arp4,
Yaf9, Eaf5
3rd round
TEL2-TAP
TTI2-TAP
5th round
4th round
2nd round
PHO23
DOT6-TAP
SNT2-TAP
Ume6
Cti6;Dot6
Ash1;Tod6
Dep1
Rxt2
Rpd3
Pho23-TAP
Sds3
Rxt3
Sap30
SIN3
Sin3-TAP
Rpd3, Ume1
RPD3
Ecm5;Snt2
Rpd3-TAP;Rxt2
UME1
Cti6
Ume1-TAP
Pho23
RCO1
Rco1-TAP
SWR1
Swr1-TAP
Bdf1;Swc2
Swc3
Rvb1;Rvb2
SWC3
Swc3-TAP
Swc4;Arp4
BDF1
Bdf1;Swr1
Bdf2
Rvb1, Rvb2
RVB1
Ino80
Arp8;Tti1
Arp5;Ies1;Bdf1
Swc3;Tel2
Rvb1-TAP
Rvb2
Act1;Pih1;Tti2
Arp4;Ies2
Ies3,Vps71
Yaf9;Swc4
Taf14
Nhp10
Ies4,5,6
Swc7;Tah1
ARP6
Swc3;Bdf1
Arp6-TAP;Swc4;Arp4
Rvb1;Rvb2

Images of Coomassie stained gels produced in immunoprecipitation experiments that deciphered the Chromatin Central environment in *S.cerevisiae.* Corresponding baits are indicated on the top of each lane. Proteins were identified by mass spectrometry as described in the Materials and Methods section, although for presentation clarity, only bands containing proteins relevant for Chromatin Central are annotated. Full list of proteins identified in each of the experiments is provided in Table S2 in Additional data file 1

IPs were performed in sequential rounds, as explained in the text. The series started with IP of ESA1-TAP and then 5 candidate subunits of plausible ESA1-containing complex (termed NuA4) were, in turn, tagged and immunopurified in Round 1. Immunoisolation of YNG2-TAP and EPL1-TAP did not bring new interactors, compared to IP of ESA1-TAP and validated the composition of the complex. At the same time, IPs of YAF9-TAP and SWC4-TAP produced new interactors that were not associated with ESA1 directly. They were subjected to tagging and immunoisolation in rounds 2 and 4. Baits for the new round of IPs were selected in the similar manner, as presented in the figure.

Gel images of immunoisolations of DOT6-TAP, ASA3-TAP and TEL2-TAP (Rounds 3 and 5, respectively) are not presented since no bands were detectable by Coomassie staining. These gels were cut in ca.30 slices that were separately digested with trypsin and analyzed by LC-MS/MS.

Gel image of the immunoisolation of SNT2-TAP (Round 5) is presented in Figure 5A

Figure S3

A

YBR095C/Rxt2 family alignment

```
ScYBR095C        26 ISTFTRRIIKEKSG---NYQVLKRSLDGKLIYPEATGISSNRGNKLL-QRSEVVTRRDLNNSK--PMIEQTVFYNGSEHRLLQTNIVTDSRRKRIKFTPDINVEPVLVGDENDIDGSEKEDENIT
CgXP_447168.1    33 INAFTRRVLKEKAG---NYRPLKRGIDGKFIYPHASGVTTNRGNKLL-QGSELISRNRLDTSR--PLEEEKVFYNGSEHRLLQ------STRKRMKFPTIPQSESVLQTLPTDDPLEEDFDE---
AgNP_983783.1    18 ILNFTKYVLSQRTG---NFPELNHTEDGT-VFPSIRGETTNRGYKLY-QDAKGVSWRRLRVDT----LEKKVFYSGSDHKLLA--------RKRMKFST----TPQFHRNDGDNDTSDTEESDDP
KlXP_452063.1    19 IKRFKRQVMVKLSG---NYPPLNQSQEGV-IFPEVRGETSNRGNKLL-QHADAVTYSKLREKE----HGSKVFYNGAEHKLLS--------RKRMKFNN------GSHRRSGKRTTADTGDESED
DhXP_459263.1    10 ISRFKQSV-------------LSKSLPG----PGPEIGATSRGNK--------ITPARTSDNSL----IPKIVDYEGSTHLVLTNDAIMKSTYRNKRRWHEMY---GERDEESDSDEYEDEDDEDS
CaXP_722833.1    10 ISRFKEALSAQKLDTDRNSKTLNEENESLPVY---------NRGLKL----SKTIQPSFNTL------RSKVTEYGGKKYSVLSNESIEIDNLHNKRKWNEIY--GDNKQDQNEDDEDDEEDDELE
PgXP_001482217.1 10 ISQFKQSLFQRRTA-----------------VQPPALVVS-NRGNKL--SSSGLGGPADGDL------RTKLVEYDNTSQVVYTNDELERSNRRSKRKWN----------AEHGVNDDDNESETSDS
YlXP_501726.1    59 SCLFCSPTTITNTV---------------SDSDESDTNISTNRGNKLKRAAEDVHMTKLPAPPL-GNHRLKIVEYNGVRRPVLY--------KRRRRDDNDDNDDDDGNNDENPDNNDDDDDSNP
AnXP_658979.1     5 AALIAD----TIVG-MKRALRNENDFSGP-DDPITQP--TNRGNKLR-GNARFVKEGAMGYIH-----AEGLYKQKIEHAGYT--------RYILHHN------PVRYDSEGDELDDDDEDSE--
SpSPBC428.06c     8 IERFKQ--------------ALFEDSDASDSDSSIGEALTNRGLKRKKGSKNVYYGCVGNSSGSSIDIDVCYYNIGNTKRGVV-------SHFRRRI--------------DPEWLDHDNPYND--

ScYBR095C           deyygeeddddLSKLVNVKEILTPILSLGDIINHKTISRTFSSPILKNLALQIILMIEKEQMSVVRYSQFLEVFLGDH---PEPIYESNLNLPSYNHNLT--[83]-NEEIESARQLSQIALQRN
CgXP_447168.1       ---------INNLSKLVNVRELLTPISSLADICKRDSIKRTFNRTILKDLALNCILMIEKEQNSVTTYSQLLDVFLGDY---PTPIYEKTLRLPKYDHSLT--[94]-LEEVENARQLVQIALQRN
AgNP_983783.1       E---------DLHDLVDVRKVLSPISSLADVATHPAVSRTFQSKVLRDLALDMILMVEKEQESVISYSRLLEVFLGDF---PDALHEEQLGLPGYDHKLK--[67]-TDQLETTRQLAQIALQRN
KlXP_452063.1       S-ADYSDGDNDLNNLVAVKEILSPISSLKDVIQHPSHSRTFGNQALKELALSNVLMVEKEQENVINYSKLLEIFLGDF---PDALYEDALKLPEYDHKLT--[60]-SDDIETTRQLAQIALQRN
DhXP_459263.1       DDE-------HPFKRLKLGEILSPLTHPSEVISHPAILKTYKLPIFNKMSYELIETIELEQNNLNWLNKLLQVLNGED---WFFLLEENLGLLKYDHGLN-[105]-QEDLIN---YLQVSVQRQ
CaXP_722833.1       YDEDKEEHPLKKSRII---ELLSPLNHPSEIISHPAISKTYKSQTLSKLASELIDLIEIEQTNLNWLNKLLQVLNGED---WFYLLEENLGLKDYDHGL---[59]-KEELIN---YLQVSIQRQ
PgXP_001482217.1    DPGSDDDHPL---KKIRLSEILAPLTHPSELVTHTAILKTYKLPVFTNLANNLIHLIEVEQNNLNWLNKLLQVLNGED---WYYVLEDKMGLEKYDHGL---[58]-REELIN---YLQVSIQRQ
YlXP_501726.1       YRG------------IQLEEILAPLTHPADLPHHKSMARTMTSTTLRTLSTRALDVICQEQKHVVQFMKLMSVFLGDD---PSYILADNMQLPDYD-------[37]-RDAAEETRQLTQIALQRC
AnXP_658979.1       ADAAVAEENPFSE--IALEHFLCPLKHPSELPSHPSLSHAYTSKALSHMTQAIEAKLRQERALLWRARNLHRQLLGDGSWAPCGIFETPEDRLIFEPQIV-[223]-PNEAEDTRRLLWSYVQKQ
SpSPBC428.06c       ---------------INIAEIMSPLTKPQDLLTHPAISSIFEQNYLSILASSALEIISAEHKYTAHLEQLMVALLGDD---PS-LPGPPHEVFGISPEQ---------CRELTI---TVQEALEKS

ScYBR095C           KEFIRNLQKIRKSVIKANRIRGRILNWSREYLGISDD 379 (420)
CgXP_447168.1       QEFIRNLQKIRGCIVKCNRIRERITSWSREYASIPEE 380 (421)
AgNP_983783.1       QEFIRNLQKIRNCIVKTNRIRERILVWGKEMAGIPED 331 (366)
KlXP_452063.1       QEFIRNLQKIRNCIIKANRIRERILMWGKEFAGISEK 331 (366)
DhXP_459263.1       QEYIKNLIQIRNGLVRAERLKQDLHKWGKEMYDKKSS 350 (350)
CaXP_722833.1       HEYIKNLISLRNGLVRADRLKSDLYKWGKEMYEKKSS 318 (318)
PgXP_001482217.1    HEYIKNLTQLRNGLVRADRLKQDLLKWAKEMHDKKST 303 (303)
YlXP_501726.1       EEFIRCITSVRMGLLRADRFRGQVYRWCKEMGDQGNE 333 (343)
AnXP_658979.1       EETVRGLEHMHESLLRACRMKEDVFEWCKAEGHVGEL 475 (516)
SpSPBC428.06c       KEFIRCWTNVRMDLLRAIRFKNKVIAYCQGEDYNGNT 228 (242)
```

B

YAL013W/Dep1 family alignment

Dep1 Similarity Region 2

```
ScYAL013W         20 DQESVLSNIDFN---------TDLNHNLNLSEYCIS--SDAGTEKMDS 56
CgXP_447071.1     24 DQESVLSNIDFNGQDGEVI--TMRSELNLSEYCVS--SDADTEKMDD 66
AgNP_984516.1     75 DKSSVLSKV-----------TSQEGDLELSELYIS--SDAETEKMEN 109
KlXP_455434.1     72 NSTSGLSKV---------TSPEHPNSVSLSDIYIS--SDAETEKMDL 108
CaXP_720932.1    129 DHDDKVPKDSNITTGDDVKNRDSYESSELSDLDEN-QSEAETDKMDF 174
DhXP_458391.1     48 GQESQENNSK---------TNRDSYESSELSDLGED-ESEAETDKMDF 85
YlXP_505054.1    115 TDDMPLPTSNGRKASLLNPADLSDDSSELSELED---SEAETERLYL 158
PgXP_001484462.1 35 GNDAGVGSVA--------GVQNATDESSELSDLGDD--SEAETDQMDF 72
AtXP_001210140.1 41 GRSSSLSEID---------DMPSDYESPKPEKLAEN-DSEAETERIED 78
SpSPBC21C3.02c   10 IPHEILPKEPF---DLPMNNLKSSPKNKDSEKRIN-NSIAESEQVVD 52
```

Dep1 Similarity Region 2

```
ScYAL013W        173 IEMEEQRMTALKEITDIEYKFAQLRQKLYDNQLVRLQTELQMCL--EGSHPELQVYYSKIAAIRDYKLHRAYQRQKYELSCINTETIATRTFIHQDFHKKVTDLRARLLNRTTQTWYDINKE
CgXP_447071.1    359 MVLEERRQQALKDITEIEHSFAELRQKLYENKLRRLETELQMCV--EGSHPELHEYYEKISKLRDFKLHRTYQRQKYELKCIDIETRATRTMIHQNFLRCVNELRSQLLQDTTTKWYDINKE
AgNP_984516.1    307 IEREKLRQDALKDIIEIEYEFAELRQRLYENEMAKLQTELQMCL--EGSHPALQTYYQKIDSIRDFKLKRAYQRQKYELECIDKETRAVRTFIHQNFYRQVSDLKHKLLNQTTQKWYDMNKE
KlXP_455434.1    372 MEKERIRQDALAELTNIEVEFAQLRQSLYENKLSKLQNELQMCL--DGSHPQLQTYYQKIASIRDFKLKRMYQRQKYELECIDTETRATRTFIHQDFYRKVSDLKHKLLGNTTQKWYDINKE
CaXP_720932.1    371 VDLNKQRNLAIQELIAIEAKFAEVRDKLFKDKLSLLEKELQLCL--DGSHPELSKIYGKINEFYQDGLRLANANLMYKLKCVDKETIATRTSIHQNFLRNLMDTKNGMITDTTSLWYKINKE
DhXP_458391.1    299 VDINEKRKLAIEELIIIESSFAELRDKLYQDKLNLLEHELQLCL--EGSHPELSRIYYKINQFHQDSLKQANSNLNYKLKCIDVSTIASRTSIHQNFLKKLMDCKNDMITDTTSLWYKINKE
YlXP_505054.1    382 ADKVDMRPAAMTSLAEIEVDFAKLRDFMFKEKLAAFEVEMGLCV--KGTHPELHAVYEQISKMRDDKIKLAKTRRKYKLQCINNQTRASRVQIHQQFLKDQGDARSGLLLKTTEEWYRVNRE
PgXP_001484462.1 282 ADMSEKRQAAVAELIEIEKAFASLRDKMYHDKLNLLEHELQLCV--EGSHPELSKIYYKINAFHQDALRQANSNLSYRLKCIDRETVACRTAVHQNFLKQVYDSKSEMITNTTSMWYKINRE
AtXP_001210140.1 307 TRTAAKRISAMESLSVLEREFATLRDKIYDERISKLNRELEMLNGPNPTHPEYLQNLEVIKRHRDAKINYEHTLFQYRIKALMNKSLAERAQLHSTYFQRVRDIREKHSSSISKQFYAIQHD
SpSPBC21C3.02c   299 DEKAIKRKEAFDALLNIETEFTFLRNRLYGKKLLKLNEHEEMIQ--NETHERFNACIDLITERRDDRVRLATENL---MKQLGN--------------IKNVMDYVTK------QRKYQLLFD

ScYAL013W            RRDMDIVIPDVNYHVPIKLDNKTLSCITGYASAAQ-LCYPGEPVAEDLACESIEYRYRANPVDKLEVIVDRMRLNNEISDLEGLRKYFHSFPGA------PELNPLRDSEINDDFHQWAQ 405 (420)
CgXP_447071.1        RRDMDIIIPDINYHVPVKTANKTLSCITGYAGPAQPRRYLGEPLSEDLECENISYRYNGNPVDKLEVIVDRMRLNNELSDLEGLKKYYHAFPGA------PSLSTLRDSEIQDDFNYLRQ 592 (592)
AgNP_984516.1        RRDMDMLVSEIGYHVPVKIANKSLSCITGYAAPAQ-LKGDGDPLPEDLECEGINFRFRNNPVDKLEVIVDRMRFNNQLSDFEGLKKFFGGFPGA------PSLNGLKDSEIYEDMQSLRE 539 (540)
KlXP_455434.1        RREMDVVVPEVNYHVPIKISNKSLSCITGYAAPAN-LRRPGDLLSEDLLCENINFRYHNNPVDKLEVIVDRMRFNNELSDLDGIRKFYGGFPGA------PELGGLKDSEIHEDLAGIQH 604 (606)
CaXP_720932.1        RNQLDQLVPDFTFAAIPSIPNGSISIEESIVNG-----------NIDGLAESSVSKKLQKQNTLIELVKQRNNINEQLGILNGLVEFHG-FPSA--------ISSSLSEEISDDQSNELL 590 (610)
DhXP_458391.1        RNQLDQLVPDYNFTAIPLIPNYTATVPIEEGAN------GVANGILNGHEIAPLSKKTIKQNTVFELVEQRNNLNCQLGILNGLLQFHG-LPSA-[15]-LLLRKATDDEINEDLRAMGI 540 (542)
YlXP_505054.1        RRAMDLMVPDYGYTI-----------------------------------------PESKSEQIHQRNAQVNEISLLSGISKYVG-FPAA------PVISQATEDETNEDLTALGL 569 (730)
PgXP_001484462.1     RNQLDQLVPDYSYSASGGAFVS--------------------------NDGNDDGSTALPQSVLYELVSQRNAINHQLGILNGLVE-FSGIPAA-[13]-LLLRKASEEEIADDLRAMGV 501 (503)
AtXP_001210140.1     RFKTDELSPQHYIPFPTRRSQQI--------------------------------------------AQQTAYNQEVSIMAGVAKYV-GFPAA------PSLSAARPSELEDDLEKMGI 496 (711)
SpSPBC21C3.02c       KRRIRQALLTKIATKCFQLLNKQKSVHD-----------------------PTYITQKTMSYRQSALLQKQRIEY--EAAVLCEL-NSFAGFPTA------PIIETASFDDIRNDLLEMGC 485 (491)
```

C

YAL011W/Swc3 family alignment

Swc3 Similarity Region 1

```
ScYAL011W          112 WVQGEMFELYWRRPKKIVSES---------TPAATESPTSGT--------------IPLIRDKMQKMCDCVMSG--GPHTF-KVRLFIL 174
CgXP_447073.1      124 WISGEMFEVYWTRANKVPSLAIKDESIIKELESSKEQDASG-------------------KDRMQRMCDCELMA--GPHTF-SIKLFIV 190
AgNP_984524.1       94 WIKAEMFELYWSKQYMNMKERER-------MLKEGIDPDDID--------------QSAAREKMHKLCDGILTG--GPHTL-PVRLFIL 158
KlXP_455443.1       77 WVKGEMFQLYWAKQYMNTKEKEE-------LKKEGIDPDSID--------------QSAAREKMNKLCDCMMQG--GPHQF-PIRLFIL 141
PgXP_001487644.1    86 WIKQSAYAKKMA-------------------AQDSKYYEKVQNDPNSDARQPILLGDVSARDVMVKLCDGTLDL--GPHTF-EIRMFIA 152
DhXP_458338.1       88 WLKQSSYAKKMQ------------------QLDQEKNTHKTQKEEKSSERVPILGNDVSARDVMVKLCDASLSM--GPHKF-EIRLFIA 155
CaXP_710630.1       87 WVKQSSYVRKLL--------------------ELDKPLPKSMENDIYLNREMVLNNDSSARDIMVKLCDGGLSFSSGIHSF-EIRIFIA 153
AnXP_661438.1      121 WLSDGILERYWT-------------------KPKKTKREQLE-------------GKNPPKESMSKVGPCNIVV--GPHLF-DAMLYTV 174
YlXP_502373.1       89 WLEGEMFDKFWTRPQRGKK-------------LAG--------------------GEVNARERMSKLCECTLSI--GPHDF-DVKLFLV 141
SpSPAC4H3.02c       69 CLNGALFEKFLP--------------------EENYKLSGGT-----------------VFSHVRYIDTATLCV--GPLRFEDTKFYFV 118
```

Swc3 Similarity Region 2

```
ScYAL011W          442 TAFQQKYVQGAEIILEYLEF-------------THSRYYLPKKSVVEFLEDTDE-------------IIISWIVI---HNSKE 495
CgXP_447073.1      482 TAFQQKYVTGAELVLEFAEN-------------TNFRFLLPKKAIIQYIPESNR-------------YKMSWLQI---HNQSD 535
AgNP_984524.1      510 TTFQQKYLNGADILFEYLEN-------------SNMRFLFPKDAILEQLENEES-------------YLMSWIVV---HNKKE 563
KlXP_455443.1      581 TTFQQKYSNGADMVFEYVEN-------------ANVRFLLPKYSILEQLEGEES-------------YLLSFIVV---HNRRE 634
PgXP_001487644.1   499 TAFQEKYVKDATVLFEFAEN-------------SNVRYALPKDAIAEVLPQTKENI-----EADEKDILFSFLWV---HNQKE 560
DhXP_458338.1      514 TAFQEKYLTDATLLFEFVEN-------------ANVRYMLPQEAVCEVLPPATYNA----EEPDNKDILVSFLWI---HNQNE 576
CaXP_710629.1      367 TAFQERYLEDATLLFEFVEN-------------PNVRFLLPKDAICEVLPASTMINAENGNNSDNRDILMSFIWI---HNQKE 433
AnXP_661438.1      538 KPPVPPRFNYKSVVFEFTSPLTPYGSSTSGHAGSGDRYLFPENTILEWLPGGTT-------------VIASFLLV---RKVDP 604
YlXP_502373.1      837 PAFLKNLPSKVIVVFEFKDN-------------PVHRYVLPQDSILEMRTESNE------------ILISTLLVLGHAHDYSD 894
SpSPAC4H3.02c      275 INNKKEVRRHFDIVMEFSDA-------------PDTKWIFPRESVLSHVFNTDKK------KLEALSLLFHVYRP---TEDRN 335
```

Swc3 Similarity Region 3

```
ScYAL011W          581 QIGTRLSGYNLWYQLDGYD   599  (625)
CgXP_447073.1      614 AVGTRLSGYNLWYQLDGYD   632  (659)
AgNP_984524.1      642 ARGRRLNGFNLWYQLDAYD   660  (688)
KlXP_455443.1      711 DRGSRLSGYNLWYQLDAYD   729  (758)
PgXP_001487644.1   767 ETGTRTSNYYLWYQVDGKQ   785  (834)
DhXP_458338.1      751 KSGTRTASFYLWYQVDGKL   769  (867)
CaXP_710629.1      637 EVGTRTSSFYLWYQVDGKL   655  (805)
AnXP_661438.1      752 DRAERAPEGFLVYRLPREQ   770  (841)
YlXP_502373.1     1074 AHGLRDEGVPVKYNIPLPV 1092 (1130)
SpSPAC4H3.02c      374 RRMSNLEEPKYYMQFHEQT   391  (391)
```

D

YNL215W/Ies2 family alignment

```
ScYNL215W        219 MLLDLLEDG-GSKK------KLTDEEIQLRRAENARKRKNLSEKRLEEEKQDTINKLLKKRAGKSRSH--LPNDDEKND---GSSSFVKPRRPYNSEGMT-RILR---RYEEDLFCTF 320 (320)
CgXP_448439.1    256 MLISILDDN-PFKK------KLTEEEIQLRRAENARKRKNLSEKRLEEEKRETLNKLLKKRAGKSRSK--VDKDEPENT-----ASTIKPRRPYNSNGMV-RIIR---KRDEDLYCIF 355 (355)
AgNP_982769.1    167 MVLDLMDEN-NVRK--RGTDHLTEEELQLRRAENARKRRNLSEKKLEEEKQDTINKLLKRRAGKSRSN--LQASEKEAS--VDEAAYSKPRRPYHAIGML-RVLR---TASEDRFAIA 273 (291)
KlXP_453916.1    232 MLENLIGLQ-AKRH--GNRKELTEEEMQLRKAETARKRKNFIEKRLEEEKQDVLNKLLKRRATKTKSDPKSINTPASNG--EDEATYSKQRRPYITAGMT-RTII---NKTGITYSLP 340 (340)
AnXP_663864.1    260 DFLQLPMEP-QIKK------HLTAEERAMRRAEMARRRKNLSEKRNEEEKMDTINRLLRKQAPKRRGR--IPAAEAAEN--AAADQEAAAETDFVDPTMV-RWIS---GREGSRVAVP 362 (385)
PsXP_001384162.1 280 QFIELDDNYTTAKKSR-LKKTETEEQTALKKAESARRRQDYKMKALEEEKRDTLNKLLKRRATKTRE---VQDKEGTVE---VVKLATKARRPVLQHPALFRYVSNITTLGGNSVLAV 390 (395)
CaXP_714448.1    262 QFIELDDSGKKAKS-[10]-EETEEEIALRRAENARKRQDYKKKVLEEEKRDTLNKLLKRRATKSREI--INEEDNKDGSDMGNNVLYKKKRPMLEHPAFIRYVNNTTSLNGNSVLSY 381 (382)
DhXP_458098.1    291 EFLELDDNMNNSKK-ARPKKTETEQQIALKKAESARRRQDYKMKVLEEEKRDTLNKLLKRRANKTRE---VDDKEGSAD---IFKLALKPRRPILDHPALTTWVSNTTTLDGNSVLRF 401 (405)
SpSPAC6B12.05c   180 ELLELPPET-SGRK------KLTPEEMALRRIENARRRKNQSERRLEEEKMETINRLLKRQSNTGKPR--RGRAPNNPT---SDSISRSKAVPKDRINMYQPFQCVRFKSTKEGSSLG 284 (295)
```

E

YOR189W/Ies4 family alignment

```
ScYOR189W           2 SQESSVLSESQEQ--LANNPKI--EDT-----SPPSANSRDNSKPVLPWDYK--NKAIEIKSFSGYKVNFTGWI------RRDVREERQRGSEF--TASDVKGSDDKATR----
CgXP_446774.1       2 SNESTI-EPDTPP--ADDRMKS--RDT---ADKDGGETHNGKKDYGKVKHWVEVEQGIPVKSFTSYTLNLSGWV----------------------------------------
AgNP_982688.1       1 ------MSQEQPP--ADPTPQASPAAP------GPKSLAPPGARPARPAHWL--AATIPVKSFSGYSLQFAGWAAD----PAAKPENANS------------------------
KlXP_452436.1      20 TAAETGKTDEQRK--ITTTDKTDDKPV-----EEPAEKKPKTSAQIKASHWK--PGSLEIKAFTGYALKLRCWKNV----DKEGPITSAVGSQA--EAKEDASIEADAET----
PgXP_001482792.1   33 QKKAAEKKTGAAS----IAGSKTSSPAPDSKSKEPNAKVTKPKSTKKAKTWV--KKPLQFKTFSGFKVKYVTWRQK----EPRKESKAKAEAKA--EAKAEKAEAKAE------
CaXP_721483.1      52 QRSSPAPEGGASATGPTISNYKVNVGL-[12]-ITSGQYSLDKSGKPAKRWV--KKPRSFKTFTG--FKVTCVSFK----PEHGPETKETQ-----KTEEKEVISNT-------
DhXP_458818.1      65 KVSSPAPSTNPEV--SNVNNFKINSGL-[12]-INSEHYLLDKSGNPTKKWI--KKPTQFKTFSGFKIKYVTWRQK----DHGKEPKNVAPPPP--AIATVKEDEN--------
YlXP_505046.1      55 ----------GSS--HTKSGPKDTDSA------INSQLRALDRTGKPPRHWV--KTPVQIKSFTNCAFDVPEWGSS----DKGMNERGEKKVKL--EVE---------------
AnXP_682369.1     154 ISEDGTSENVRLS--THRLGPKANTGA------INAGLRALDRSGTPCRRWE--RKPLQLKSFTGVQWQLPSWRAPRTQTQEPNGETKEGVLETGDSDSRANQSASGA-[11]-
SpSPAC23G3.04      78 LAESSKPSSGAAT--PTRSAPKSSAGL------INSGLRALDRSGKPCRRWE--KKPISIRSISTIVWKLPLWIGT----PDSIPNTPELPVKT--TLDSVNEIAAAL------

SpSPAC23G3.04         STHAESSPMDATSPVDSMPESAT 192 (194)
ScYOR189W             KKEP---ADEDPEVKQLEKEGED 112 (116)
CgXP_446774.1         ------REDQKKEVKDERKDTSD  84  (85)
AgNP_982688.1         ---------AQPKP-TGPRPPAAA  84  (84)
KlXP_452436.1         SIEPQEKGDSKPDQDESQSESET 137 (143)
PgXP_001482792.1      AERAEKDKADAEVNETPETKDNT 151 (179)
CaXP_721483.1         STNGTTTP--GNTPAVSLAPNNE 172 (172)
DhXP_458818.1         TAGSSTAPMSASETPETKLET-- 187 (187)
YlXP_505046.1         ----------------------- 127 (127)
AnXP_682369.1         STPAHPSMVDASSPAIAMAA--- 282 (282)
```

F

YPR085C/Asa1 family alignment

```
ScYPR085C          1 MRGFSNEIILKRTLTLSDFTLRYHKRGITALQVIKAP--SVSNVPVLLSGDNYGYFVMWDLVTKRPITHIEIEGNSHIIAFWWVETT-----NVLYILSKDSMLRIFELDSSTQLSIDLVRKLSQ
KlXP_453467        1 ----------------MPFTLRYHTCGVTSLLHWND----EQDVPTLISGDEKGSILVWNLLSRKPYYKYTCRG--QIVSFQQLND-------LIIATSKDHTLRILKFP--STLTT-----KEG
AgNP_983713        1 ----------------MRFTLRAHVSTVTDLKPVS-----HRQTPHLLSADSKGCLYLWNLISRRPIASIDLKT--HIIAIEVVGQG------LYAALARDNKLRFISLQEESRLTR-----IND
CgXP_448565        1 ------------MERAATHFLRLHKSGISALCSGVIDPDYGITSPVLFSGDIDGEVTIWNLITRRPIFTSKICN--EQVVDIQFLEG-----KYLSLLCKDHKLRLYELLKLGAIVK-----QSD
PgXP_001481942.1   1 ------------MEPRQKGALRGHKNPITCTIVFKDH---KRNCNTLVTSDNEGWVAWWDISTKRPLGVWRAHS--NSILSVVQIDH-----NLLLTHGKDSCVRIWAITQYEGFSKTFPAENHT
DhXP_459853.1      1 ------------MKPIELCTLRGHLNDITCTEPY-----YIGGKVSLVSADSNGWIIWWDINTRRPNCVWKGHD--SNIVTLRQICN-----GLLLTHSKDSDIKIWDVENFKSGSREMPAEKYN
CaXP_719157.1      1 -------------MLSTKFTLRSHKSSVTYIYQD------PRTPFNLFTADSSGLIINWDLAIRRPKKSWQAHT--DTILTISTIH------NHLLTHSRDNTIKIWD-----------------
YlXP_500459.1      1 --------MDLSGAPVPVALLRGHTHPVTSLRFYNA---------FLVSGDESGWVFWWSLVTRRPLAIWKAHHE-AILSLVWMDET-----HLL-TQGRDDKLYVWRLELDAQGKSGLSVKPPS
AfXP_753542.1      2 SEPSEVTNQAARPPATPIYILRGHAAPIHALHLY-------NQNLRLISADADGWVIVWDLVMKRPVAAWKAHEG-AILEVKGSTSA-[7]-YTCPRHGRDHKLRVWRFRRQDEEVLQKTLPVEI
SPAC1006.02        1 -----------MVVPTPFYVLRGHSSSVTSVLFDA--------NEYLYSGDEAGFVICWCLTSMRPKCAWRAHTK-TILGMQIVKG------GALCTHGRDCRLVTWKIDFNCMTDNFMSLSKLA

ScYPR085C            ------ANKTDHLQWTKIYE-MPINTLNFANFIIEA-[10]-YRLVCCHTDDSETIDIYQIIEDSTF-----KLKRPFNNINFPRFL----KQQNFLGIS-----------KDSKFGIIMRFAKL
KlXP_453467          ------FPDYSLPELELIYE-IPVNTLNFANTAVEQ--[5]-YRLWCCNTQDSETIDIYEFDLRDSK-----SLKRIHRALSLYEVI----SGLVDPSTM-----------KFDKLGTTMKFIIY
AgNP_983713          ------KVSRELQSLEIVYE-IPVNCLNFANFALQD--[5]-YRLWCCNTMDAESIDVYEFQLGDRQ-----SFKRTFNAINLFDSV----AGLAEAKQKF----------RFDKMGIVMRFIVA
CgXP_448565          ------FGGGDKVDLKQIFE-VPVNTLNFANYVLTY--[5]-FELVTCHTQDAHFIDIYEFETPELN-----SLKRFSKAIDFLPML----RDRFGDNLLP----------KMDGLGIIMKFYKV
PgXP_001481942.1     ------ESG--SHRWPEYVE-IPVNTLNFCNVCYLN------GKLITPATQDSNNFDIYSIFPASAA-[9]-SLRRIVANADPLALHKKAKSKENLTMGIDFEISDENS--KRDGFGIMMKVVFV
DhXP_459853.1        -[19]-NELLEAFPLPENVV-IPVNALNYCNVDYSN------HHLITPATTDSNNFDLYLIFKPSHQ-[9]-NLKRVAANIDPWKLYKKTITQLNKEQGVEFEIGNENDILKRDKFGIMMKVLFV
CaXP_719157.1        ------------ESYICILE-IPCNALNFSNICIIY------DLLITPASINSNNLDVYKIDKDWQ-------ITRLISDFDVYKLVNKGEIIEEIGSSGT----------SRNDFGIIMQMKII
YlXP_500459.1        --[4]-DDPTDYPKPWLTYS-LTVNSLNFCQVAWVN------GLLAKPDLDSSDKVELLEWTDGA--------FRVVWNDI-YPRL-------------------------NGIKTGIVMDLNIM
AfXP_753542.1        --[3]-PQANTASQPWLVHS-LPVNALNFCAFSPLF-[17]-ESADATPQEAPRSPALIAVPNA---------LNSGAIDL-FHLP------LERRVCTIPAD--------TTTDTGMVMAVHLF
SPAC1006.02          -[10]-SETEKSSAFISIHSNIVVNSLTFCPFSYSP--[2]-KIVVLCNTLNFEELDVYDDESLYHP-[9]-RLQTRIQPE-ESVG----------------------------KTGSVMSTSVT

ScYPR085C            ND-------VIFLGYENGFVVGFKIT---------FDEGLQRDI-AELVHVSNDHYPNPILDMCVSGDE--LYSCSTDDFITKYKIPVNL------QLETKYLRDDALLIKCPSSLRVSEPSKVH
KlXP_453467          EG-------IIYLGFESGFVIGLRLTQG-----------------LHIVYISSANYPEPVLDLTVGKDLGKVISSSTNSSLGLHTPNINT------ESHNNVSSKDVVVDDST-----IHSEMVN
AgNP_983713          GD-------TVFCGYESGVVVGLRIRDK--[1]------------LQICYASFAHYPEPVLSLAHDRTERRVFSSSTTDQVCVHNIPTPD------LPVVTSVSGIQVVANAG-----TREEVIN
CgXP_448565          NE-------VVYCGFESGYVIAFRRYRN-[35]-SSVNDLELDNVIEIVLVDRAHYPDPVLDMAPNPKKNGIICSSTTNKLVLTSVDEQY-[9]--FKSDEYLLDKKNCLLMSTNLKIDGSAVKD
PgXP_001481942.1     SPT------LFYIGYESGHIIGFSLTEY-[15]-FDASFINKEPTLEIIYISAFHCPHPITALFFHDK---LYAGSAGKTLSIH------------TKQSSIAEEPEI---------------ES
DhXP_459853.1        RDD------LFYIGYESGHLIGYHIDFS-[34]-FDKTIINKDPHIKMIYMNDSCSPYPIISLVYDNKENKIICGSAGKQLTFHKIPEEF---------SQFNDISDC---------------KR
CaXP_719157.1        TTN-[12]-IIYVGFESGDIVGLQLILP-[11]-NDKTLINQSAKFILRYHNSTHAPNPVICLSNLDSV--LVSGSTTNKVIIHSDPI-----------------------------------EI
YlXP_500459.1        NK-------KLIVGYEGGAVAVFDIS---------DRNRYTPV--LN-YYVVSHVQPVLSVRAHPTKKE--FVSSSADSLIVKHPIKDQV-[39]-RGFDVPGLELEEGIEVKEEEKPESKPLDA
AfXP_753542.1        TGP--[4]-YVASAYEDGHVMLFARRGL--[6]-LQGTTASWKW-EKLYACRPHSQPVLSIDVFPQGNY--FLSSSADALLVKHPIPRLS------ECSHQSTNRPEEAVLKTV---------NT
SPAC1006.02          VTD--[4]-LLAAGYESGHVVQYICSLE--[3]-TVTLDFKAVW-KMVYAYKSHSQPVLSVEYAGSK----LFSTGADDCICLHPTPSSI------ADDLGSLPHPIFR--------------KT

ScYPR085C            LPLKNIGHIDKVKD---DYLVVSSWSG-MTIVYNMRTSEVEQ-----TFVKSKNNLVVSDS-[44]-LRLGQLRRIKALAKCNWCLIGYEDGTIKLNKI-------- 443 (443)
KlXP_453467          IPINKVSHIQQVDN----LLIAASWSG-RTVVFDINGNKVLA-----SILKERGQVLIDDN-[32]-TSEGTRRRWIRFYKETWCLIGYADGSIKATEVTFDNLITA 399 (399)
AgNP_983713          APLKKIGHLAVLND----ILLLTSWHG---YVLGLQDQKELF-----RYRKERNLLCVDDS-[78]-LPIGGHRRLKNFLEHKWYITGYNDGTITVSQI-------- 395 (395)
CgXP_448565          MMCKNIGFVLSLGD----YIITGNWSG-KTYIGRIESDKAVL-----AAAKSRSLIEVNES-[42]-LTPGKLRRLNAFVQSKWYFIGYTDGTIGLYRAE------- 475 (475)
PgXP_001481942.1     YNLKIAGVQDIAVL--PELAIVAFWNGVVKGYEKQSTEAESD-[6]-DFSTEPNTISQFKP-[53]----------ARQVSPRLLTVGYADGVTIMYEI-------- 449 (449)
DhXP_459853.1        YNLRHSGIQSVSIN--NSLLVVGFWDGLIKGYDLDL-NELF------KYCKRLPRIDVLES-[36]-SLIKHK---RDITTTNLLISSYYDGTITIFKV-------- 476 (476)
CaXP_719157.1        MKMDHSGIQAIVNF-KNDRLIFGYWNGYI-QYGDISINQ--------SLPKLGN--------[32]-SKYSVLLKSKRNLVSPLLLAGYEDGSILAYNI-------- 377 (377)
YlXP_500459.1        VNVRHSGLSSLQLDSDGDLIMTAGWDGKVRLFTYDDISKV-------SVFHEREGVGCVA--[12]--------RLAKALTTRWIAVGGKDGKIELYTI-------- 410 (428)
AfXP_753542.1        KHAGQQGLRIR---SDGKIFATAGWDSRIRVYSCKTMKEL-------AVLKWHKEGCYTI--[44]-LAAVQHQRNRKVQTTHWLAAGSKDGKISLWDI-------- 459 (460)
SPAC1006.02          KHCGQQNIRIR---SDNKILATAGWDGRGRVYSCQTLAPL-------AVLKYHSDGINS----------------LAFHPGSNVIALASKDTRISLWKLY------- 368 (368)
```

Figure legend for Figure S3

A: Multiple sequence alignment of fungal Rxt2 family members

Budding yeast Rxt2 detected the *S. pombe* protein SPBC428.06c at the 1st PSI-BLAST iteration with an E-value of 0.062, which decreased in the 2nd iteration to 2E-10. The *S. pombe* orthologue of Rxt2 picked up the budding yeast Rxt2 protein at the 2nd PSI-BLAST iteration with an E-value of 2E-05 (Supplemental Table 1) at which point the two proteins show a reciprocal best hit relationship. All Rxt2 orthologues share an N- and C-terminal region of sequence similarity with a long central gap that lacks significant sequence similarity.

B: Multiple sequence alignment of fungal Dep1 family members

Budding and fission yeast orthologues of Dep1 detected each other with significant E-values at the 2nd PSI-BLAST iteration (Supplemental Table 1) and show a reciprocal best hit relationship. Following a short region of similarity in the N-terminus of Dep1 family members, the proteins contain a central region without significant similarity that is followed by a highly conserved C-terminal part. In the *S. pombe* sequence, an SDS3 domain is detected in the C-terminal conserved region.

C: Multiple sequence alignment of fungal Swc3 family members

S. cerevisiae Swc3 did not pick up the *S. pombe* orthologue in PSI-BLAST searches due to profile drifting that result from extended low-complexity regions present in Swc3 protein sequences. The *S. pombe* SPAC4H3.02c protein however detected *S. cerevisiae* Swc3 the 2nd PSI-BLAST iteration as the best hit in *S. cerevisiae* with an E-value of 3E-05 (Supplemental Table 1) upon inclusion of the *Y. lipolytica* orthologue for PSSM construction. Conserved regions of Swc3 family members are restricted to 3 short regions in the very N-terminal and C-terminal parts of the proteins.

D: Multiple sequence alignment of fungal Ies2 family members

Both, *S. cerevisiae* and *S. pombe* Ies2 orthologues detected each other in the 1st PSI-BLAST iteration with a significant E-value (Supplemental Table 1). The conserved region of this protein family is limited to the very C-terminal region of the proteins.

E: Multiple sequence alignment of fungal Ies4 family members

S. cerevisiae Ies4 did not detect the predicted *S. pombe* SPAC23G3.04 due to profile drifting induced by extended low-complexity regions of Ies4 family members. SPAC23G3.04 did however pick up the orthologue of *S. cerevisiae* Ies4 as its best hit. *K. lactis* Ies4 also showed a reciprocal best hit relationship with both, the *S. cerevisiae* and *S. pombe* Ies4 proteins. Even though the conservation of Ies4 members is very remote, they share a central region of similarity with a distinct pattern of charged and aromatic amino acids.

F: Multiple sequence alignment of fungal Asa1 family members

S. cerevisiae Asa1 detected *S. pombe* SPAC1006.02 as the best hit in fission yeast in the 2nd PSI-BLAST iteration with an E-value of 4E-28. The *S. pombe* protein SPAC1006.02 does not detect Asa1 as the best hit, which is mostly due to repetitive alignments with alternative WD40 repeat proteins from budding yeast that result in lower E-values. It shows however a best reciprocal hit relationship with for example the *A. fumigatus* orthologue of Asa1.

Additional information for multiple sequence alignments in Figure S3

Identification, Expect values (E-values) and PSI-BLAST iterations required for identification of remote orthologues of *Sc* and *Sp* complex components

S. cerevisiae	*S. pombe*	PSI-BLAST iteration	E-value	PSI-BLAST iteration (RB)	E-value (RB)
YBR095C (Rxt2)	SPBC428.06c	1 (2)	0.062 (2E-10)	2	2E-05
YAL013W (Dep1)	SPBC21C3.02c	2	4E-17	2	1E-36
YAL011W (Swc3)	SPAC4H3.02c	-	-	2	3E-05
YNL215W (Ies2)	SPAC6B12.05c	1	4E-12	1	6E-11
YOR189W (Ies4)	SPAC23G3.04	-	-	2 (*K. lactis*)	0.060
YPR085C (Asa1)	SPAC1006.02	2	4E-28	1 (*A. fumigatus*)	3E-34

Abbreviations of organisms used in multiple sequence alignments

Abbreviation	Organism
Sc	*Saccharomyces cerevisiae*
Sp	*Schizosaccharomyces pombe*
Cg	*Candida glabrata*
Ag	*Ashbya gossypii*
Kl	*Kluyveromyces lactis*
Dh	*Debaryomyces hansenii*
Ca	*Candida albicans*
Pg	*Pichia guilliermondii*
Yl	*Yarrowia lipolytica*
An	*Aspergillus nidulans*
At	*Aspergillus terreus*
Af	*Aspergillus fumigatus*

Figure S4

A

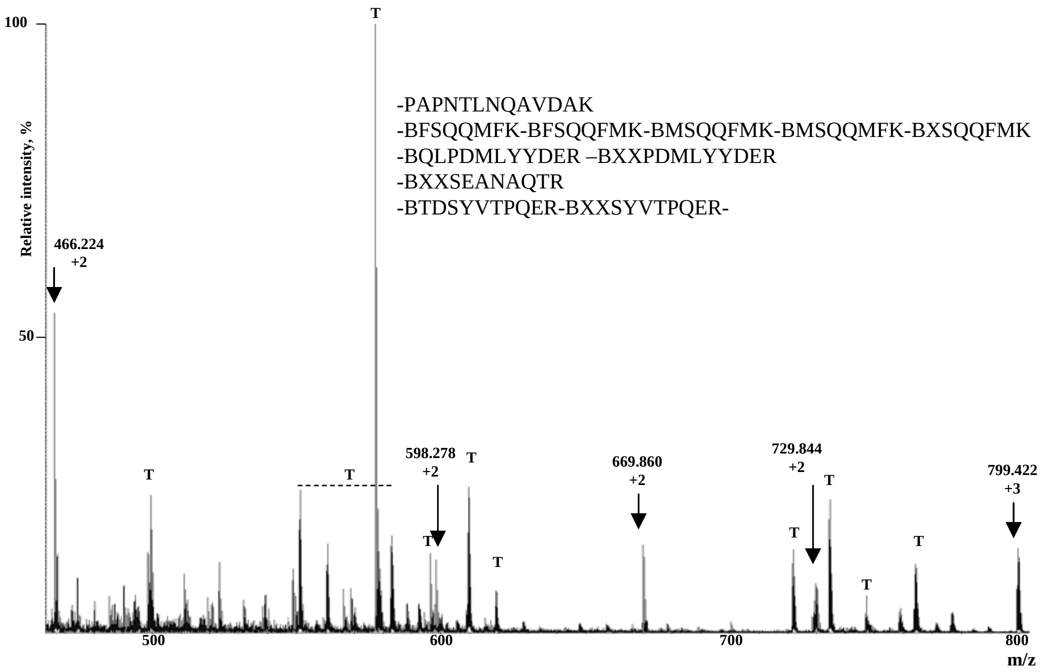

B

MAAQKKQGERVLPARSTRKRR**QLPDMLYYDERTDSYVTPQERSLSEANAQTRPAPNTINQ AVDAK**QSAREARVQELMKSKLYHLRKQQKQARHKRDQWAIDYLEWKKNEDEDVWNSDAE ATGPAEHTSSFLDALR**FSQQFMK**AE

C

Chromosome I

```
Frame +2
Query:       1 MAAQKKQGERVLP 13
Sbjct: 3314150 MAAQKKQGERVLP 3314188

Frame +3
Query:      14 ARSTRKRRQLPDMLYYDERTDSYVTPQERSLSEANAQTRPAPNTINQAVDAK
Sbjct: 3314241 ARSTRKRRQLPDMLYYDERTDSYVTPQERSLSEANAQTRPAPNTINQAVDAK

QSAREARVQELMKSKLYHLRKQQKQARHKRDQWAI 101
QSAREARVQELMKSKLYHLRKQQKQARHKRDQWAI 3314501

 Frame +2
Query:     102 DYLEWKKNEDEDVWNSDAEATGPAEHTSSFLDALRFSQQFMKAE 145
Sbjct: 3314549 DYLEWKKNEDEDVWNSDAEATGPAEHTSSFLDALRFSQQFMKAE 3314680
```

Identification of Iec6p - a new protein with unknown sequence in *S.pombe* – using mass spectrometry and similarity searching. **A**: MS spectrum acquired from intensive Coomassie band running at ca 20kDa on Sp_Rvb1p-TAP IP gel (*see corresponding gel pic*.); trypsin autolysis products are marked with "T". 5 peptide precursors were fragmented from the sample; however MASCOT search against a complete protein database with uninterpreted MS/MS data did not produce any confident hits. Fragmentation spectra of five peptide precursors (indicated with arrows) were manually interpreted, then all sequence suggestions were merged in one string and searched against protein database and *S.pombe* genome using MS BLAST similarity searching program (peptide query is shown in the insert). When no confident hits were found in a protein DB, four out of five peptides were matched exactly to a 132 aa-long non-coding region of the chromosome I. **B**: Iec5p final sequence. The new protein was cloned and all five peptides suggested by mass spectrometry were found in the final sequence (underlined in bold). **C.** *IEC5* gene contains two introns.

Figure S5

Plausible molecular architecture of the Chromatin Central environment in humans inferred from the published compositions of individual complexes (see Additional data file 12). Note that PCAF complex [103] (presumably orthologous to yeast SAGA / SLIK) was not included in this table since it was not characterized by us. N-CoR2 protein was found in HDAC3 complex and also interacts with HDAC1/2 proteins [90].

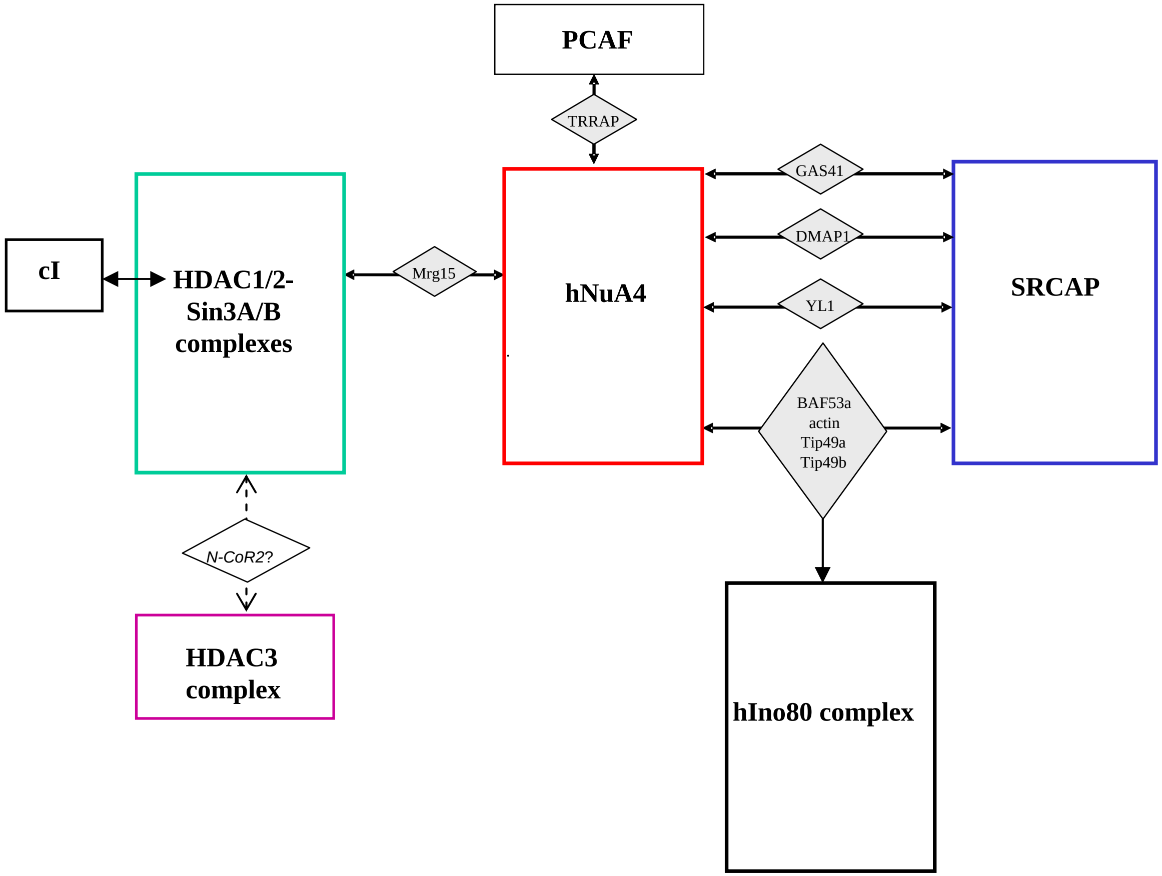

Table S5

Domain annotation of the members of Chromatin Central proteomic environment

Proteins sequences were extracted from the NCBI Reference Sequence database and subjected to local InterProScan searches [99]. Identified domains, their position within the sequence and corresponding *E*-values were automatically extracted.

Domain ID	Domain name	Occurrence		Domain details		
		SC	SP	ORF	Position	E-value
**NuA4 histone acetyltransferase complexes**						
IPR000357	HEAT	1	0	YHR099W	2182-2217	0.6
IPR000403	PI3_PI4_kinase	1	1	YHR099W<br>SPAC1F5.11c	3400-3676<br>3310-3587	1.1E-69<br>3.1E-76
IPR001487	Bromodomain	2	1	YLR399C<br>SPBC21D10.10	153-242/ 320-409<br>190-277	4.9E-32/ 6.5E-40<br>1.8E-20
IPR001965	PHD	1	1	YHR090C<br>SPAC3G9.08	224-271<br>230-277	2.30E-16<br>2.00E-13
IPR002110	Ankyrin repeat	1	0	YHR099W	477-3255	0.014
IPR002717	MOZ_SAS	1	1	YOR244W<br>SPAC637.12c	220-406<br>237-423	1.5E-137<br>0
IPR003151	PIK-related, FAT	1	1	YHR099W<br>SPAC1F5.11c	2756-3108<br>2622-2976	2.9E-128<br>0
IPR003152	PIK-related, FATC	1	1	YHR099W<br>SPAC1F5.11c	3712-3744<br>3623-3655	1.10E-12<br>3.70E-10
IPR004000	Actin	2	2	YFL039C<br>YJL081C<br>SPBC32H8.12c<br>SPBP23A10.08	6-375<br>15-488<br>1-375<br>6-433	2.2E-272<br>1E-22<br>0<br>1.70E-48
IPR005033	YEATS	1	1	YNL107W<br>SPAC17G8.07	40-123<br>38-121	5.30E-41<br>2.40E-37
IPR006562	HSA	1	1	YDR359C<br>SPCC1795.08c	347-418<br>467-539	1.30E-29<br>1.60E-32
IPR008676	MRG	1	1	YPR023C<br>SPAC23H4.12	86-379<br>45-324	6.40E-145<br>0
IPR009057	Homeodomain-like (SANT)	2	2	YDR359C	644-708	2.90E-06

				YGR002C<br>SPAC9G1.13c<br>SPCC1795.08c	154-220<br>100-152<br>704-764	4.70E-09<br>6.34E-04<br>0.0
IPR011616	bZIP_1	1	1	YML007W<br>SPAC1783.07c	63-126<br>81-138	0.00085<br>5.40E-04
IPR012423	CT20	1	1	YNL136W<br>SPBC16A3.19	8-135<br>36-130	1.70E-62<br>2.20E-45
SSF48371	ARM repeat	2	0	YHR099W	5-476/3256-3699	1.00E-10/3.70E-08
SSF53383	PLP-dependent transferases	1	0	YNL107W	195-219	0.013
IPR013910	PAP1	1	1	YML007W<br>SPAC1783.07c	274-648<br>250-551	8.6E-188<br>0.0
IPR000953	Chromo domain	2	2	YOR244W<br>YPR023C<br>SPAC637.12c<br>SPAC23H4.12	33-86<br>64-113<br>34-86<br>19-72	5.9E-06<br>1.1E-07<br>1.1E-08<br>4.4E-08

Rpd3S/Clr6S histone deacetylase complexes

IPR000286	Hist_deacetyl	1	1	YNL330C<br>SPBC36.05c	20-331<br>7-318	2.30E-183<br>0
IPR001965	PHD	2	3	YMR075W<br>SPAC16C9.05<br>SPAC2F7.07c	262-309/416-470<br>119-166<br>265-312/408-455	3.40E-17/ 1.8E-05<br>9.70E-14<br>6.50E-13/5.80E-04
IPR002017	Spectrin repeat	1	0	YOL004W	455-923	0.00054
IPR003822	PAH	3	3	YOL004W<br>SPAC23C11.15	239-285/426-472/679-725<br>54-100/160-206/271-317	8.40E-16/3.70E-15/2.20E-12<br>4.90E-10/3.40E-10/4.20E-10
IPR008676	MRG	1	1	YPR023C<br>SPAC23H4.12	86-379<br>45-324	6.40E-145<br>0
IPR011046	WD40	1	1	YPL139C<br>SPAC29A4.18	169-443<br>7-412	4.60E-21<br>5.51E-33
IPR013194	HDAC_interact	1	1	YOL004W<br>SPAC23C11.15	747-848<br>333-434	1.30E-72<br>7.7E-62
SSF46942	Elongation factor TFIIS domain 2	1	0	YMR075W	544-651	2.50E-18
IPR000953	Chromo domain	1	1	YPR023C<br>SPAC23H4.12	64-113<br>19-72	1.1E-07<br>4.4E-08

Rpd3L/Clr6L histone deacetylase complexes

IPR000679	GATA	1	0	YKL185W	499-536	5.90E-11
IPR001138	Zn_clus	1	0	YDR207C	769-807	1.90E-09
IPR001965	PHD	2	2	YNL097C<br>YPL181W<br>SPBC1685.08<br>SPBC1709.11c	282-329<br>74-123<br>50-103<br>250-297	3.60E-13<br>1.80E-14<br>6.20E-12<br>2.60E-11
IPR002017	Spectrin repeat	1	0	YOL004W	455-923	0.00054
IPR000286	Hist_deacetyl	1	1	YNL330C<br>SPBC36.05c	20-331<br>7-318	2.30E-183<br>0
IPR001202	WW_Rsp5_WWP	0	1	SPBC12C2.10c	71-98	0.401
IPR003822	PAH	3	6	YOL004W<br>SPBC12C2.10c<br>SPBC1734.16c	239-285/426-472/679-725<br>200-246/367-413/528-574<br>133-179/274-320/425-470	8.40E-16/3.70E-15/2.20E-12<br>3.90E-15/3.20E-10/6.20E-10<br>6.20E-12/2.90E-10/2.10E-09
IPR009057	Homeodomain-like (SANT)	4	0	YBL054W<br>YER088C	71-124/435-482<br>68-121/392-443	1.40E-09/0<br>3.90E-10/0
IPR011046	WD40	1	1	YPL139C<br>SPAC29A4.18	169-443<br>7-412	4.60E-21<br>5.51E-33
IPR013194	HDAC_interact	1	2	YOL004W<br>SPBC12C2.10C<br>SPBC1734.16C	747-848<br>592-693<br>492-592	1.30E-72<br>2E-66<br>1.5E-50
IPR007526	SWIRM	0	2	SPAC14C4.12c<br>SPCC1682.13	207-297<br>182-272	2.20E-22<br>5.20E-20
SSF47762	PAH2 domain	1	0	YBR095C	195-236	0.0078
IPR013907	Sds3	2	1	YIL084C<br>YAL013W<br>SPBC21C3.02c	30-71<br>191-232<br>317-358	7.4E-14<br>1E-16<br>2.6E-08
IPR013904	Rxt2-N	1	1	YBR095C<br>SPBC428.06c	61-221<br>32-158	1.4E-87<br>8.2E-63
IPR013951	Rxt3	1	1	YDL076C<br>SPCC1259.07	139-253<br>220-301	1.2E-61<br>6.1E-38

Swr1 chromatin remodeling complexes

IPR000330	SNF2_N	1	1	YDR334W<br>SPAC11E3.01c	699-999<br>450-746	3.60E-115<br>1.70E-105
IPR001487	Bromodomain	2	2	YLR399C	153-242/320-409	4.90E-32/6.50E-40

				SPCC1450.02	89-178/259-348	1.60E-31/1.00E-37
IPR001650	Helicase_C	1	1	YDR334W<br>SPAC11E3.01c	1281-1359<br>1026-1104	7.40E-24<br>5.10E-24
IPR003959	AAA	1	1	YDR190C<br>SPAPB8E5.09	74-121<br>66-113	1.60E-07<br>6.80E-07
IPR001965	PHD	0	3	SPAC343.11c	298-345/1173-1220/1456-1505	0.38/2.00E-08/1.30E-04
IPR003349	JmjN	0	1	SPAC343.11c	90-128	0.0085
IPR013129	JmjC	0	1	SPAC343.11c	512-628	1.00E-39
IPR004000	Actin	3	3	YFL039C<br>YJL081C<br>YLR085C<br>SPBC32H8.12c<br>SPBP23A10.08<br>SPCC550.12	6-375<br>15-488<br>4-432<br>1-375<br>6-433<br>159-400	2.20E-272<br>1.00E-22<br>1.50E-07<br>0<br>1.70E-48<br>1.00E-28
IPR004198	zf-C5HC2	0	1	SPAC343.11c	716-768	4.80E-18
IPR005033	YEATS	1	1	YNL107W<br>SPAC17G8.07	40-123<br>38-121	5.30E-41<br>2.40E-37
IPR006562	HSA	1	0	YDR334W	340-411	5.60E-24
IPR007529	zf-HIT	0	1	SPBC29A3.05	98-127	6.80E-10
IPR008895	YL1	1	1	YDR485C<br>SPBP35G2.13c	708-737<br>8-233	4.50E-15<br>7.20E-82
IPR013272	YL1-C	1	1	YDR485C<br>SPBP35G2.13C	708-737<br>269-298	1.3E-15<br>1.89E-10
IPR009057	Homeodomain-like (SANT)	1	1	YGR002C<br>SPAC9G1.13c	154-220<br>100-152	4.70E-09<br>6.34E-04
IPR010339	TIP49	2	2	YDR190C<br>YPL235W<br>SPAPB8E5.09<br>SPBC83.08	126-449<br>122-433<br>118-441<br>119-430	4.70E-235<br>1.80E-220<br>0<br>0
IPR011421	BCNT	1	1	YBR231C<br>SPCC576.13	218-292<br>141-213	9.90E-42<br>1.20E-36
SSF53383	PLP-dependent transferases	1	0	YNL107W	195-219	0.013

Ino80 chromatin remodeling complexes

IPR000330	SNF2_N	1	1	YGL150C<br>SPAC29B12.01	709-1017<br>845-1144	7.50E-109<br>2.70E-103
IPR001650	Helicase_C	1	1	YGL150C<br>SPAC29B12.01	1334-1412<br>1464-1542	7.20E-24<br>2.50E-24

IPR003959	AAA	1	1	YDR190C<br>SPAPB8E5	74-121<br>66-113	1.60E-07<br>6.80E-07
IPR004000	Actin	5	5	YFL039C<br>YJL081C<br>YNL059C<br>YOR141C<br>SPBC32H8.12c<br>SPBP23A10.08<br>SPBC365.10<br>SPAC664.02c	6-375<br>15-488<br>38-420/612-747<br>842-878<br>1-375<br>6-433<br>19-211/589-716<br>527-614	2.20E-272<br>1.00E-22<br>3.40E-08/6.10E-11<br>2.50E-05<br>0<br>1.70E-48<br>9.10E-26/1.70E-11<br>8.20E-05
IPR005033	YEATS	1	0	YPL129W	29-113	1.30E-40
IPR006880	PAPA-1	1	1	YNL215W<br>SPAC6B12.05c	237-317<br>198-293	6.80E-06<br>4.30E-53
IPR007087	zf-C2H2	0	1	SPAC144.02	106-131	2.80E-05
IPR009071	HMG-box	1	1	YDL002C<br>SPAC10F6.08c	87-163<br>202-254	4.00E-18<br>2.82E-05
IPR010339	TIP49	2	2	YDR190C<br>YPL235W<br>SPAPB8E5.09<br>SPBC83.08	126-449<br>122-433<br>118-441<br>119-430	4.70E-235<br>1.80E-220<br>0<br>0
IPR013175	DUF1711	0	1	SPAC23G3.04	1-194	7.60E-119
IPR013272	YL1_C	1	1	YEL044W<br>SPAC222.04c	114-143<br>66-95	1.70E-14<br>3.20E-15

ASTRA complexes

IPR000357	HEAT	1	2	YHR099W<br>SPBP16F5.03c	2182-2217<br>1098-1134/1574-1612	0.6<br>0/0
IPR000403	PI3_PI4_Kinase	1	1	YHR099W<br>SPBP16F5.03c	3400-3676<br>3358-3631	1.10E-69<br>3.50E-09
IPR003151	PIK-related, FAT	1	1	YHR099W<br>SPBP16F5.03c	2756-3108<br>2682-3036	2.90E-128<br>7.70E-118
IPR003152	PIK-related, FATC	1	1	YHR099W<br>SPBP16F5.03c	3712-3744<br>3667-3699	1.10E-12<br>0.19
IPR002110	Ankyrin Repeat	1	0	YHR099W	477-3255	0.014
IPR003959	AAA	1	1	YDR190C<br>SPAPB8E5	74-121<br>66-113	1.60E-07<br>6.80E-07
IPR010339	TIP49	2	2	YDR190C<br>YPL235W<br>SPAPB8E5.09<br>SPBC83.08	126-449<br>122-433<br>118-441<br>119-430	4.70E-235<br>1.80E-220<br>0<br>0

IPR011046	WD40	1	1	YPR085C<br>SPAC1006.02	17-443<br>4-367	5.00E-15<br>8.30E-20
SSF48371	ARM repeats	3	2	YHR099W<br>YKL033W<br>SPAC458.03<br>SPCC622.13c	5-476/3256-3699<br>537-945<br>550-623<br>87-952	1.00E-10/3.70E-08<br>4.80E-15<br>0.0077<br>8.80E-13

Snt2 complex

IPR000286	Hist_deacetyl	1	-	YNL330C	20-331	2.30E-183
IPR001025	BAH	1	-	YGL131C	121-259	9.90E-33
IPR001606	ARID	1	-	YMR176W	182-294	6.90E-52
IPR001965	PHD	3	-	YGL131C<br>YMR176W	319-369/1040-1097<br>1240-1290	2.80E-12/1.40E-10<br>1.40E-10
IPR009057	Homeodomain-like (SANT)	1	-	YGL131C	553-605	5.00E-06
IPR013129	JmjC	1	-	YMR176W	509-678	2.40E-37

Set3 histone deacetylase complexes

IPR000253	FHA	1	0	YLR183C	118-194	3.90E-11
IPR000286	Hist_deacetyl	1	1	YGL194C<br>SPAC3G9.07c	27-340<br>27-339	1.20E-183<br>0
IPR001214	SET	1	1	YKR029C<br>SPAC22E12.11c	315-463<br>207-344	4.70E-28<br>3.10E-22
IPR001965	PHD	1	1	YKR029C<br>SPAC22E12.11c	119-166<br>5-51	7.00E-15<br>1.20E-12
IPR002110	Ankyrin repeat	1	0	YIL112W	316-636	2.40E-50
IPR002130	Pro_isomerase	1	0	YDR155C	3-162	1.20E-116
IPR013720	LisH	1	1	YBR103W<br>SPCC1235.09	6-32<br>4-30	3.2E-07<br>5.9E-06
IPR003000	SIR2	1	0	YOL068C	208-419	1.10E-113
IPR007137	DUF348	1	0	YCR033W	361-415	7.80E-15
IPR007654	DUF592	1	0	YOL068C	40-207	1.80E-133
IPR009057	Homeodomain-like (SANT)	2	1	YCR033W<br>SPAC22E12.19	666-718/885-937<br>248-294	3.00E-09/4.00E-10<br>0.00589
IPR011046	WD40	1	1	YBR103W	147-479	1.50E-48

SPCC1235.09	246-555	1.06E-30

Table S7

Composition of orthologous complexes comprised in the Chromatin Central environment in *S.cerevisiae*, *S.pombe* (as determined in this work) and *H.sapiens* (as published by other groups).

Gene name	ORF	ORF	Gene name	GenBank ID
**NuA4 acetyltransferase complexes**				
S.cerevisiae		***S.pombe***	**Human orthologous assembly** [81,86]	
Tra1	YHR099W	SPAC1F5.11c	TRRAP	NP_003487.1
Epl1	YFL024C	SPCC830.05c	Epc1 and Epc2	NP_079485.1, NP_056445.2
Vid21	YDR359C	SPCC1795.08c	P400	NP_056224.2
Eaf3	YPR023C	SPAC23H4.12	Mrg15 and MrgX	NP_996670.1, NP_036418.1
Arp4	YJL081C	SPBP23A10.08	Baf53a	NP_004292.1
Swc4	YGR002C	SPAC9G1.13c	DMAP1	NP_001029196.1
Yng2	YHR090C	SPAC3G9.08	Ing3	NP_061944.2
Act1	YFL039C	SPBC32H8.12c	Actin	
Esa1	YOR244W	SPAC637.12c	Tip60	NP_874369.1
Yaf9	YNL107W	SPAC17G8.07	Gas41	NP_006521.1
Bdf1	YLR399C	SPBC21D10.10	Brd8	NP_631938.1
Eaf7	YNL136W	SPBC16A3.19	MRGBP	NP_060740.1
Eaf6	YJR082C		Eaf6	NP_073593.2
Yap1	YML007W	SPAC1783.07c		
Eaf5	YEL018W			
			Tip49a	NP_003698.1
			Tip49b	NP_006657.1
			YL1	NP_005988.1
**Swr1 chromatin remodeling complexes**				
S.cerevisiae		***S.pombe***	**Human orthologous assembly SRCAP** [83,86]	
Swr1	YDR334W	SPAC11E3.01c	SRCAP	NP_006653.1
Vps72	YDR485C	SPBP35G2.13C	YL1	NP_005988.1
Vps71	YML041C	SPBC29A3.05	ZNHIT1	NP_006340.1
Swc4	YGR002C	SPAC9G1.13c	DMAP1	NP_001029196.1
Arp6	YLR085C	SPCC550.12	Arp6	NP_071941.1
Arp4	YJL081C	SPBP23A10.08	BAF53a	NP_004292.1
Rvb1	YDR190C	SPAPB8E5.09	Tip49a	NP_003698.1
Rvb2	YPL235W	SPBC83.08	Tip49b	NP_006657.1
Yaf9	YNL107W	SPAC17G8.07	Gas41	NP_006521.1
Act1	YFL039C	SPBC32H8.12c	Actin	
Bdf1	YLR399C	SPCC1450.02		
Swc5	YBR231C	SPCC576.13		
Swc7	YLR385C			
Swc3	YAL011W	SPAC4H3.02c		
		SPAC343.11c		
**INO80 chromatin remodeling complex**				
S.cerevisiae		***S.pombe***	**Human orthologous assembly hINO80** [91]	

Ino80	YGL150C	SPAC3G6.12	hIno80	NP_060023.1
Arp4	YJL081C	SPBP23A10.08	BAF53a	NP_004292.1
Arp5	YNL059C	SPBC365.10	Arp5	NP_079131.3
Arp8	YOR141C	SPAC664.02c	Arp8	NP_075050.3
Act1	YFL039C	SPBC32H8.12c	Actin	
Rvb1	YDR190C	SPAPB8E5.09	Tip49a	NP_003698.1
Rvb2	YPL235W	SPBC83.08	Tip49b	NP_006657.1
Ies2	YNL215W	SPAC6B12.05c	PAPA-1	NP_112578
Ies4	YOR189W	SPAC23G3.04		
Ies6	YEL044W	SPAC222.04cp	C18orf37	NP_001092287.1
Nhp10	YDL002C	SPAC10F6.08c		
Taf14	YPL129W			
Ies1	YFL013C			
Ies3	YLR052W			
Ies5	YER092W			
		SPAC144.02		
		SPCC1259.04		
		Non-annotated gene*		
			Amida	NP_037474.1
			NFRKB	NP_006156.2
			MCRS1	NP_001012300.1
			FLJ90652	NP_775889.1
			FLJ20309	NP_060229.3
**Set3 histone deacetylase complexes**				
S.cerevisiae		***S.pombe***	**Human orthologous assembly HDAC3** [84, 85]	
Set3	YKR029C	SPAC22E12.11c		
Snt1	YCR033W	SPAC22E12.19	NCOR1 and NCOR2	NP_006302.2, NP_006303.2
Sif2	YBR103W	SPCC1235.09	TBL1XR1 and TBL1X	NP_078941.2, NP_005638.1
Hos2	YGL194C	SPAC3G9.07c	HDAC3	NP_003874.2
Tos4	YLR183C			
Cph1	YDR155C			
Hst1	YOL068C			
Hos4	YIL112W			
			GPS2	NP_004480.1
**Subunits of HDAC complexes**				
S.cerevisiae Rpd3S/L		***S.pombe* Clr6S/L** [38]	**Multiple overlapping HDAC1/2-Sin3A/B complexes, selected subunits** [87,88,105-107]	
Rpd3	YNL330C	SPBC36.05C	HDAC1 and HDAC2	NP_004955.2, NP_001518.1
Eaf3	YPR023C	SPAC23H4.12	MRG15	NP_996670.1
Ume1	YPL139C	SPAC29A4.18	RbAp46 and RbAp48	NP_002884.1, NP_005601.1
Rco1	YMR075W	SPAC2F7.07c & SPAC16C9.05	Phf21A<br>Phf12	NP_057705.2<br>NP_001028733.1
Sin3	YOL004W	SPBC12C2.10C & SPBC1734.16C SPAC23C11.15	Sin3A<br>Sin3B	NP_056292.1<br>NP_056075.1
Sds3	YIL084C	SPAC25B8.02	Sds3	NP_071936.2
Pho23	YNL097C	SPBC1709.11c	Ing1	NP_005528.3
Cti6	YPL181W	SPBC1685.08		
Rxt2	YBR095C	SPBC428.06c		
Rxt3	YDL076C	SPCC1259.07		
Sap30	YMR263W			
			Sap30 and Sap30L	NP_078908.1, NP_003855.1
Dep1	YAL013W	SPBC21C3.02c		
Ume6	YDR207C			

Ash1	YKL185W			
		SPAC14C4.12c & SPCC1682.13		
Dot6<br>Tod6	YER088C<br>YBL054W			
**Demethylase/HDAC assemblies**				
S.cerevisiae		***S.pombe***	**Human cI complex** [108]	
Rpd3	YNL330C	SPBC36.05C	HDAC1 and HDAC2	NP_004955.2, NP_001518.1
Ecm5	YMR176W		Lsd1	NP_055828.2
Snt2	YGL131C		Rcor1	NP_055971.1

* new gene in *S.pombe* found in this work
